# Cytological, genetic, and proteomic analysis of a sesame (*Sesamum indicum* L.) mutant *Siyl*-*1* with yellow–green leaf color

**DOI:** 10.1007/s13258-019-00876-w

**Published:** 2019-11-01

**Authors:** Tong-Mei Gao, Shuang-Ling Wei, Jing Chen, Yin Wu, Feng Li, Li-Bin Wei, Chun Li, Yan-Juan Zeng, Yuan Tian, Dong-Yong Wang, Hai-Yang Zhang

**Affiliations:** 1 State Key Laboratory of Crop Genetics and Germplasm Enhancement, Nanjing Agricultural University, Nanjing, 210095 China; 2 Henan Sesame Research Center, Henan Academy of Agricultural Sciences, Zhengzhou, 450002 China

**Keywords:** Sesame, 2-DE, Proteomic, Yellow–green leaf mutant, Photosynthesis

## Abstract

**Background:**

Both photosynthetic pigments and chloroplasts in plant leaf cells play an important role in deciding on the photosynthetic capacity and efficiency in plants. Systematical investigating the regulatory mechanism of chloroplast development and chlorophyll (Chl) content variation is necessary for clarifying the photosynthesis mechanism for crops.

**Objective:**

This study aims to explore the critical regulatory mechanism of leaf color mutation in a yellow–green leaf sesame mutant *Siyl*-*1*.

**Methods:**

We performed the genetic analysis of the yellow-green leaf color mutation using the F_2_ population of the mutant *Siyl*-*1*. We compared the morphological structure of the chloroplasts, chlorophyll content of the three genotypes of the mutant F_2_ progeny. We performed the two-dimensional gel electrophoresis (2-DE) and compared the protein expression variation between the mutant progeny and the wild type.

**Results:**

Genetic analysis indicated that there were 3 phenotypes of the F_2_ population of the mutant *Siyl*-*1*, i.e., *YY* type with light-yellow leaf color (lethal); *Yy* type with yellow-green leaf color, and *yy* type with normal green leaf color. The yellow-green mutation was controlled by an incompletely dominant nuclear gene, *Siyl*-*1*. Compared with the wild genotype, the chloroplast number and the morphological structure in *YY* and *Yy* mutant lines varied evidently. The chlorophyll content also significantly decreased (*P *< *0.05*). The 2-DE comparison showed that there were 98 differentially expressed proteins (DEPs) among *YY, Yy,* and *yy* lines. All the 98 DEPs were classified into 5 functional groups. Of which 82.7% DEPs proteins belonged to the photosynthesis and energy metabolism group.

**Conclusion:**

The results revealed the genetic character of yellow-green leaf color mutant *Siyl*-*1*. 98 DEPs were found in *YY* and *Yy* mutant compared with the wild genotype. The regulation pathway related with the yellow leaf trait mutation in sesame was analyzed for the first time. The findings supplied the basic theoretical and gene basis for leaf color and chloroplast development mechanism in sesame.

**Electronic supplementary material:**

The online version of this article (10.1007/s13258-019-00876-w) contains supplementary material, which is available to authorized users.

## Introduction

Photosynthesis is widely considered as the basis of growth, development and yield in plants. Chlorophyll (Chl) is the primary photosynthetic pigment for light harvesting and driving electron transport in the reaction centers in higher plants. Leaf color mutants are helpful for systematically investigating the mechanisms of photosynthesis and photosynthetic pigment biosynthesis in plants, as the leaf colors of mutants are closely related to chlorophyll synthesis or photosynthesis efficiency (Yang et al. 2014). Many reports have shown that most leaf color related mutagenesis is involved in the structure and function variation of the chloroplasts (Li et al. 2018a, b; Zhang et al. 2006a, b), chlorophyll biosynthesis and degradation mechanisms (Oda-Yamamizo et al. 2016), photosynthesis (Slattery et al. 2017), and chloroplast development (Sugliani et al. 2016; Leister 2003; Sang et al. 2010).

There are 5 leaf color mutation types in plants, i.e., albino, yellow, light-green, stripe, and spot (Awan et al. 1980; Falbel and Staehelin 1994; Gustafsson 2010). Moreover, the leaf color mutants related with the chlorophyll variation are comprised of two categories: (1) mutants lack of chlorophyll b, such as the barley mutants lacking chlorophyll b (Thornber and Highkin 2010); and (2) mutants with reduced synthesis capacity of the total chlorophyll and chlorophyll *b*. Most mutants currently belong to the second category (Zhang et al. 2006a, b).

Leaf color can be controlled by both nuclear and cytoplasmic inheritance and presents as a quantitative or qualitative trait (Nasyrov 1987). Some leaf color mutants are caused by monogenic mutation. The recessive mutations are predominant and have a lower dominant mutation rate (Nagata et al. 2005). Cytoplasmic inheritance accounts for a small proportion of leaf color mutants (Allen et al. 1988). There are very few leaf color mutations that are of the nucleo-cytoplasmic interaction type (Murray and Kohorn 1991). At present, research on leaf colors is focused on genetic analysis (Li et al. 2018a, b) and the regulatory mechanism of leaf color in plants under stress environments (Yu et al. 2016; Huang et al. 2017b).

Sesame (*Sesamum indicum* L.) is the ‘queen of the oil seed crops’ for the high oil content (45–63%) and numerous beneficial minerals, antioxidants, and multivitamins in the seeds (Anilakumar et al. 2010; Zhang et al. 2019). However, sesame is a low yield crop, compared with the cereals and other oilseed crops. To improve the photosynthesis and increase the photosynthesis transferring frequency is the key objectives of high-yield sesame breeding. During creating new breeding materials in the past decade, we obtained a leaf color mutant *Siyl*-*1* from the sesame EMS mutant library induced through EMS mutagenesis (Wang et al. 2017). The mutant exhibited the inheritable yellow-green leaf color trait under different growing environments. Elementary selfing pollination results of the mutant *Siyl*-*1* showed that there are three phenotypes in the progeny of the mutant *Siyl*-*1*, i.e., light-yellow leaf color with seedling lethal phenotype (named *YY*), yellow-green leaf color (*Yy*) and normal green leaf color (*yy*) (the wild type). Thus, in order to systematically analyze and parse out the molecular regulatory mechanism of yellow leaf mutants in sesame, we performed the cytology, genetics, and proteomics analyses of the progeny of the yellow-green mutant *Siyl*-*1*. However, there is no study reporting a powerful approach to identify and isolate different proteins by two-dimensional gel electrophoresis (2-DE) in sesame.

In this study, we presented the main results of the yellow-green mutant *Siyl*-*1* in (1) the genetic analysis of the yellow-green leaf color trait within the progeny of the mutant *Siyl*-*1*, (2) the cytological characteristics comparison of the light-yellow leaf color mutant genotype (*YY*) and the yellow-green leaf genotype (*Yy*) and the wild type with the normal green leaf color (*yy*), and (3) the protein expression profiles of the three genotypes of the *Siyl*-*1* progeny. The specific expressed proteins related to photosynthesis in the mutant were also analyzed for the first time. The findings provided a foundation for further sesame proteomics research.

## Materials and methods

### Plant materials

The yellow–green leaf mutant *Siyl*-*1* used in this study was induced from var. ‘Yuzhi 11’ using EMS mutagenesis by the Henan Sesame Research Center (HSRC), Henan Academy of Agricultural Sciences (HAAS), China. Three chinese sesame varieties, i.e., Yuzhi 4, Zhengtaizhi 1, and Zhengheizhi 1 were used as the wild type with normal green leaf color for cross hybridization and the genetic and cytological analyses. All the above materials are available from the sesame germplasm reservoir of HSRC, HAAS.

To explore the genetic characteristics of the leaf color trait in the mutant *Siyl*-*1*, the self-pollution and the cross hybridization of 6 groups (Table 2S) were performed using the above materials during 2014–2016. The leaf color traits of each sample were observed three times during seedling stage, budding stage and flowering stage. Chi square tests (*P *< 0.05) were used to determine the segregation significance for the leaf color characteristics.

The healthy seeds of the three genotypes of the mutant *Siyl*-*1* progeny were cultured in artificial growth chambers (GPJ-400, Changzhou, China) at 30 °C with 70% relative humidity and a 14 h light/10 h dark cycle for 1 week for leaf harvest. Three biological replicates were set.

### Chlorophyll content measurement

After germinating, 3 g of cotyledons per genotype from the mutant *Siyl*-*1* progeny was collected for chlorophyll content measurement. Chlorophyll content was measured and calculated according to the methods of Arnon (1949) using a spectrophotometer (TU-1810, Beijing, China). The chlorophyll of three plantlets was extracted and measured in 3 biological replicates.

### Cytological observation

After germinating, 2 × 2 mm sections of the young leaves of the three genotypes (*yy, Yy,* and *YY*) were collected and immediately prefixed in 3% precooled glutaraldehyde fixative (pH = 7.2). Samples were rinsed with 0.1 mol L^−1^ phosphate buffer (pH = 7.2), fixed in 1% osmium acid solution, and then rinsed using the same buffer. All fixed samples were dehydrated, impregnated, embedded, and polymerized with the epoxy resin Epon-812. Sectioning was performed using a Power Tome-XL ultrathin microtome, and the samples were double-stained with uranyl acetate and citrate. The chloroplast structure was observed and imaged under a Hitachi H-7650 transmission electron microscope (Hitachi, Tokyo, Japan). For each sample, three biological replicates were observed.

### Protein extraction and 2-DE

After germinating, 3 g of cotyledons per genotype of the mutants was collected and frozen with liquid nitrogen for subsequent protein extraction. Total proteins in leaves were extracted using trichloroacetic acid (TCA)/acetone mixture (Parker et al. 2006). The optical density of the protein samples was measured at 595 nm using Bradford’s (1976) method. Samples were then stored at − 70 °C for 2-DE electrophoresis. Three biological replicates per sample were performed.

2-DE was performed using the methods of Ma et al. (2012). The 100 mL samples were mixed with 400 μL hydrated sample buffer. SDS-PAGE was performed following the method of Sui et al. (2015). Polyacrylamide gels were stained, washed, and then scanned with a Molecular Imager Pharos FX System (Bio-Rad) (AB Company, Milwaukee, USA).

### Protein in-gel digestion and MALDI-TOF-TOF/MS analysis

Target protein spots were individually cut from the gel and washed three times with ultra-sterilized water. Each protein sample was decolorized with 50 μL of ammonium bicarbonate (100 mmol L^−1^ NH_4_HCO_3_). After the excess solution was discarded, 100% acetonitrile was added for 5 min. Then, 5 μL of ammonium bicarbonate solution (50 mmol L^−1^ NH_4_HCO_3_) with 10 ng of trypsin was added per sample tube. Each tube was then covered with 20 μL of ammonium bicarbonate solution and digested at 37 °C for 16 h. Finally, 5 μL of 5% trifluoroacetic acid was added per tube and left for 10 min to terminate the reaction.

After digestion, 1 μL of supernatant was placed on the sample target point. A 1 μL aliquot of α-cyano-4-hydroxycinnamic acid matrix solution was placed on the same point. The target sample was then subjected to matrix-assisted laser desorption/ionization-time of flight mass spectrometry (MALDI-TOF-TOF/MS). Mass spectrometry parameters were set as follows: primary MS molecular weights ranged from 700 to 3500 Da, and secondary MS molecular weights ranged from 40 to 1050 Da.

### Protein identification and data analysis

Incorporating MS and secondary MS data, raw data files were converted to *.mgf files. The file was submitted to the MASCOT search engine for protein retrieval (http: http://www.matrixscience.com). The NCBI nr database was used for the collection of protein information with the taxonomic parameter set to green plants. The function of DEPs was interpreted using the UniProt database (http://www.uniprot.org). To better understand the biological function and interaction of DEPs, a protein–protein interaction network (PPI) was predicted using the online analysis tool STRING 9.0 (http://string-db.org).

### Quantitative real-time PCR analysis

The gene sequences for these proteins were obtained from the genome database of Yuzhi 11 (data unpublished) and quantitative real-time PCR (qRT-PCR) analysis. Total RNA was extracted from sesame leaves of all three phenotypes with an RNA extraction and purification kit (Sangon Biotech, Shanghai, China) according to the manufacturer’s instructions. The total RNA was reverse transcribed into cDNA with a Revert Aid First Strand cDNA Synthesis Kit (Thermo Scientific, Vilnius, Lithuania). qRT-PCR was performed with FastStart Essential DNA Green Master (Roche, Mannheim, Germany) using the Realplex 4 MasterCycler real-time PCR System (Eppendorf AG, Hamburg, Germany). qRT-PCR was conducted in accordance with the manufacturer’s instructions. The reaction conditions were as follows: 1 cycle of 50 °C for 2 min; 1 cycle of 95 °C for 10 min; 40 cycles of 95 °C for 15 s; 40 cycles of 60 °C for 20 s; and 40 cycles of 72 °C for 20 s. Three independent biological replicates per sample were performed. *SiTUB* was used as an internal reference gene to normalize the relative gene expression (Wei et al. 2013). The relative gene expression was calculated using the 2^−∆∆Ct^ method (Livak and Schmittgen 2001). The primer sequences of the genes of DEPs for qRT-PCR are shown in Table 1S.

### Statistical analysis

The data are presented as the mean ± standard deviation for three independent replicates. Data were analyzed statistically by one-way analysis of variance (ANOVA) with SPSS-17 statistical software (SPSS Inc., Chicago, IL, USA). Significant differences were determined at *P *< 0.05.

## Results

### Genetic analysis of the leaf color mutant *Siyl*-*1*

**Fig. 1:**
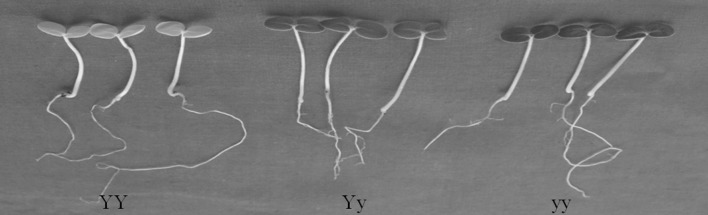
Three phenotypes of the yellow leaf mutant *Siyl*-*1* in sesame. *YY*, light-yellow (lethal); *Yy*, yellow–green; and *yy*, normal green

To clarify the genetic background of the yellow-green leaf color trait in the mutant *Siyl*-*1*, we investigated the leaf color phenotype of the self-pollinated progeny of *Siyl*-*1* (Fig. 1; Table 2S). There were three phenotypes in the self-pollinated progeny, i.e., light yellow color (named *YY*), yellow green color (*Yy*), and the normal green color (*yy*), with the expected separation ratio of 1 (*YY*): 2 (*Yy*): 1 (*yy*) using χ^2^ tests. Interestingly, the genotype *YY* would die 1 day after emergence. For the genotype *Yy*, the leaf color maintained yellow-green during the whole life cycle. The self-pollinated progeny of *Yy* presented the isolation of the leaf color trait. Meanwhile, the progeny of the genotype *yy* was normal with green leaf color and reflected the recessive homologous trait character (data not shown). Furthermore, we chose 1 line with *Yy* genotype and 3 lines with *Yy* genotype and performed the 3 crosses and the 3 reciprocal crosses (Table 2S). Phenotype investigation indicated that the leaf color trait within the F_1_ lines of the 3 crosses and the reciprocal crosses presented the expected ratio of 1 (yellow-green, *Yy*): 1 (green, *yy*) by χ^2^ tests. The genetic analysis results proved that the mutant *Siyl*-*1* (*Yy*) is heterozygous, and the yellow leaf trait is controlled by an incompletely dominant nuclear gene named *Siyl*-*1* in sesame.

### Leaf chlorophyll content and cell ultrastructure variation in *Siyl*-*1*

In order to understand the cytogenetic characters of the leaf color mutant, we firstly evaluated the variation of the chlorophyll components of the above three genotypes at the cotyledon stage (Table 1). Compared with the normal content (0.51 mg g^−1^) in *yy* seedlings, the chlorophyll a content in *YY* and *Yy* seedlings was significantly reduced to 0.01 mg g^−1^ and 0.29 mg g^−1^, respectively. Meanwhile, the content of chlorophyll b also decreased from 0.18 mg g^−1^ (*yy*) to 0.02 mg g^−1^ (*YY*) and 0.08 mg g^−1^ (*Yy*), respectively. The total chlorophyll content of the three genotypes was 0.68 mg g^−1^ (*yy*), 0.37 mg g^−1^ (*Yy*), and 0.03 mg g^−1^ (*YY*), respectively. Compared with the wild genotype (*yy*), the contents of chlorophyll a, chlorophyll b, and the total chlorophyll in the *Yy* type decreased significantly (*P *< 0.05). However, the carotenoid content of *Yy* genotype was 0.09 mg g^−1^ and insignificant differed from that (0.10 mg g^−1^) of *yy* genotype.

**Table 1:** Chlorophyll content of the three genotypes of the *Siyl*-*1* progeny during cotyledon expanding stage

Genotype	Chlorophyll a content (mg g^−1^)	Chlorophyll b content (mg g^−1^)	Carotenoid content (mg g^−1^)	Total chlorophyll content (mg g^−1^)
*YY*	0.01 ± 0.00^c^	0.02 ± 0.00^c^	0.05 ± 0.00^b^	0.03 ± 0.00^c^
*Yy*	0.29 ± 0.01^b^	0.08 ± 0.00^b^	0.09 ± 0.01^a^	0.37 ± 0.01^b^
*yy*	0.51 ± 0.00^a^	0.18 ± 0.01^a^	0.10 ± 0.00^a^	0.68 ± 0.01^a^

*YY*: Homozygous mutant (lethal) with light-yellow leaf color. *Yy*: Heterozygous mutant with yellow-green leaf color. *yy*: Wild type with the normal green leaf color

The lowercase letters within the columns indicate the significant difference at *P *< 0.05

**Fig. 2:**
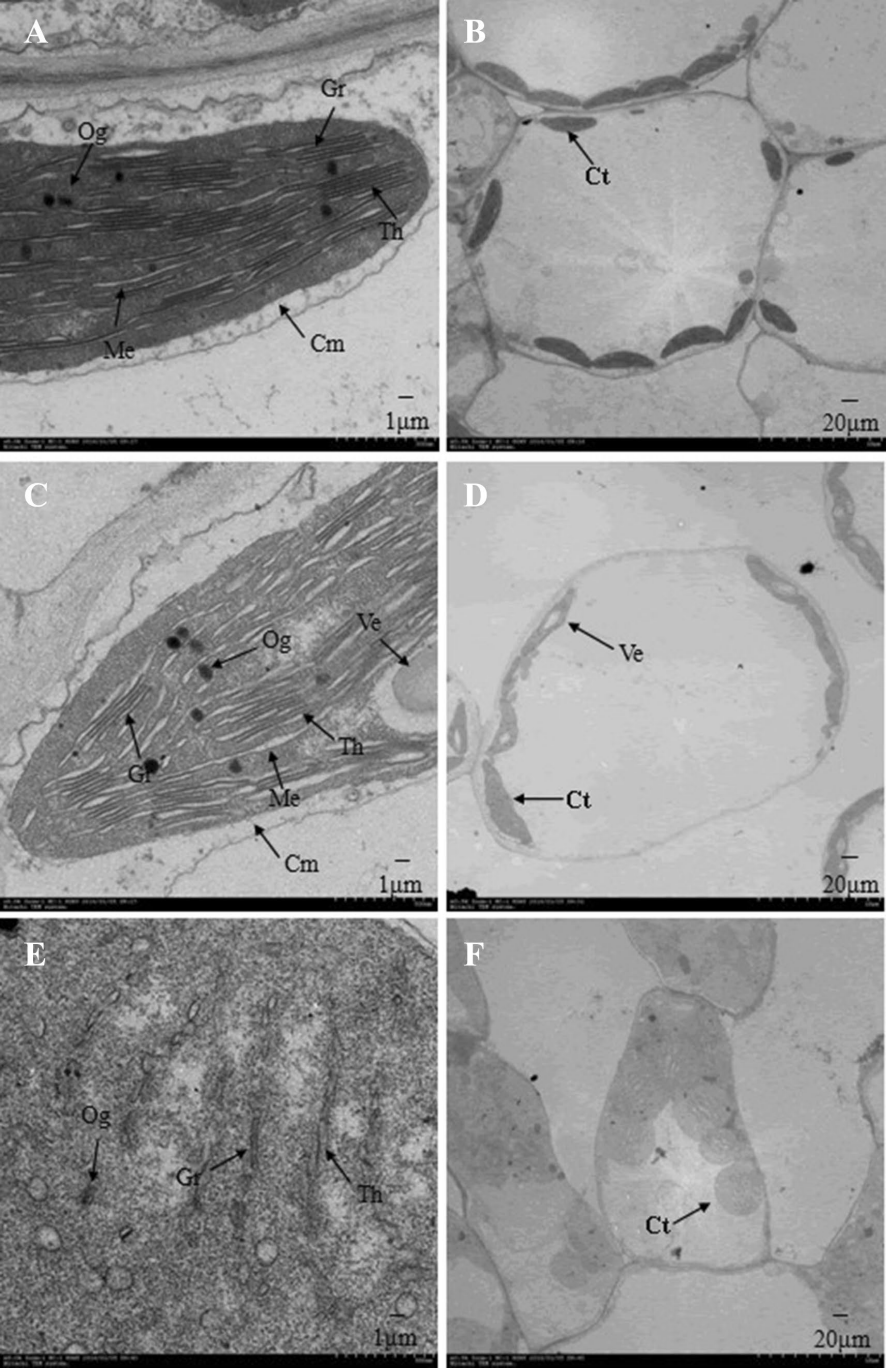
Morphological comparison of chloroplast cells of three genotypes of *Siyl*-*1*. **a** and **b***yy*. **c**, **d***Yy*. **e**, **f***YY*. *Cm* chloroplast outer membrane, *Th* thylakoid, *Og* osmiophilic globule, *Gr* grana, *Me* mesenchyme, *Ve* vesicle, *Ct* chloroplast. *YY*: homozygous mutant (lethal) with light-yellow leaf color. *Yy*: Heterozygous mutant with yellow-green leaf color. *yy*: wild type with the normal green leaf color

Meanwhile, we compared the ultrastructure characters of the leaf cells of the three genotypes (Fig. 2). As to the wild progeny (*yy*) of *Siyl*-*1*, the chloroplasts were normal with a spindle shape (Fig. 2a, b). The lamellar structure was clear and tightly folded. No starch grains presented, while a small amount of osmiophilic granules could be observed in leaf cells (Fig. 2a, b). For the genotype *Yy*, the chloroplasts were large in size with the thick spindle shape and cavities. In addition, gaps were observed in the stacking and folding lamellar structure. The chloroplast structure was abnormal and osmiophilic granules appeared (Fig. 2c, d). For *YY* genotype, the shape of chloroplasts changed from the convex lens-like shape to the circular shape. The lamellar structure was unclear and disordered and appeared as a loose strip. The thylakoid volume decreased, while the osmiophilic granules increased (Fig. 2e, f).

### Comparative proteomics analysis of the three genotypes

**Fig. 3:**
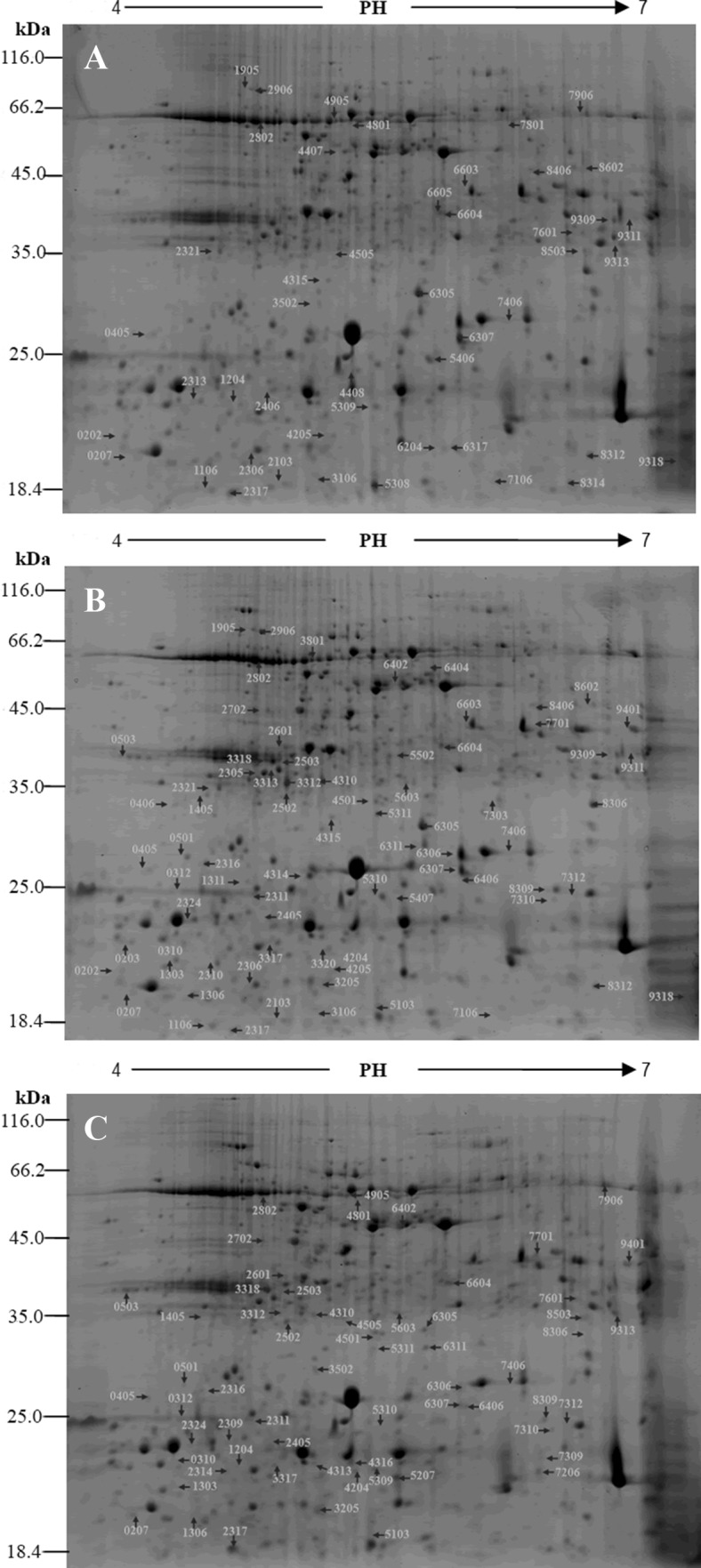
Identification of 98 protein spots according to 2-DE and MALDI-TOF-TOF MS analysis. The proteins are identified and compared among the three genotypes of the leaf color mutants during the seedling stage. **a***YY* genotype; **b*Yy*** genotype; **c*yy*** genotype. The DEPs are numbered in the images

**Fig. 4:**
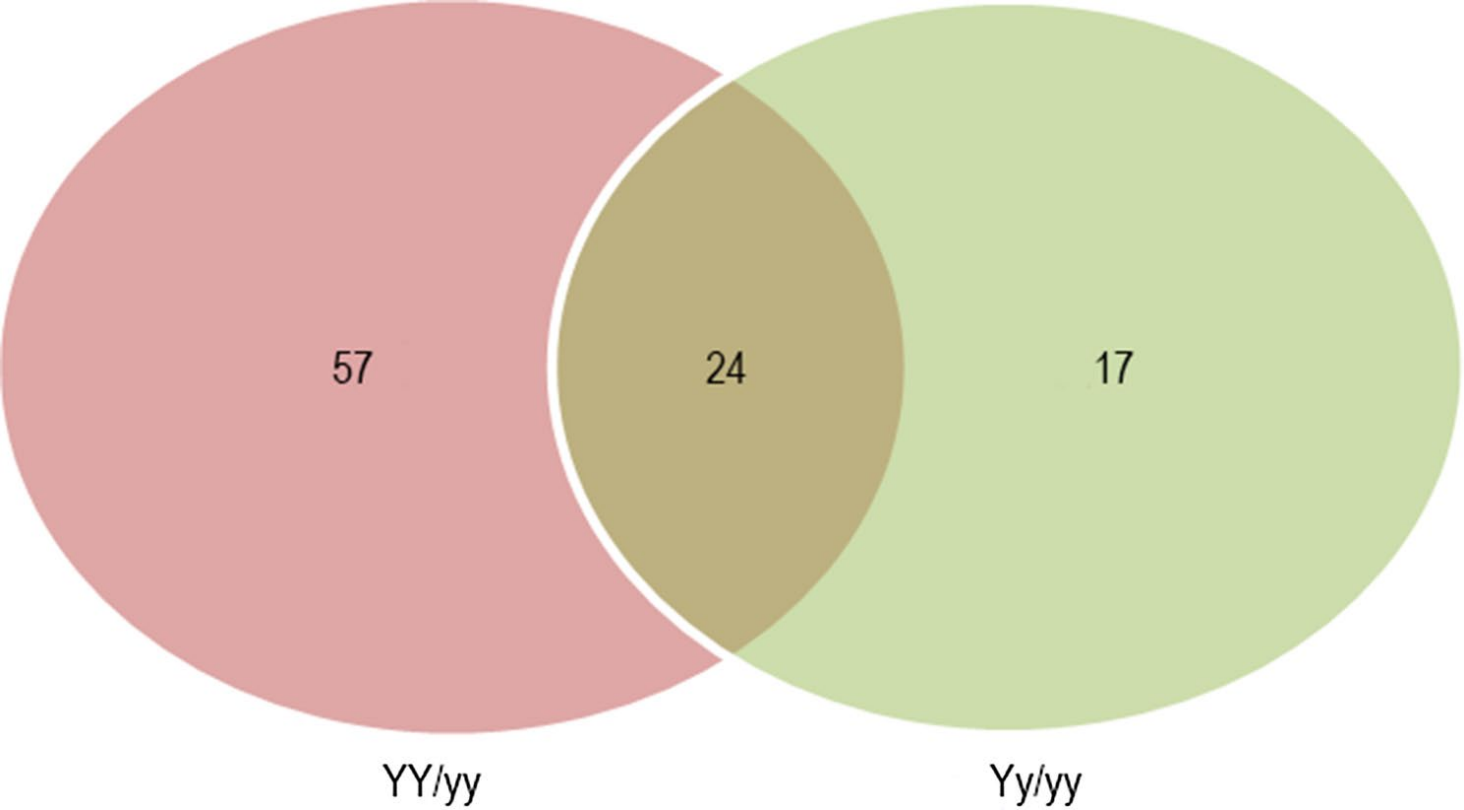
Venn diagram of the DEPs comparison of the *YY* and *Yy* mutant genotypes with the wild type

To further explore the regulatory mechanism of leaf color trait in sesame, we performed a 2-DE of the proteins in the mutant leaves. The proteomic difference between the wild type (*yy*) and the two mutant types (*YY* and *Yy*) of *Siyl*-*1* was compared and analyzed (Table 3S). A total of 518, 521, and 535 protein spots were obtained from the individuals of *YY*, *Yy* and *yy*, respectively. The isoelectric point of the protein spots mainly aggregated with the pH range from 4−7, and the molecular weight generally ranged from 10-110 kDa. Protein gel image of light-yellow (*YY*), yellow-green (*Yy*) and normal green (*yy*) phenotypes were analyzed in PDQuest 7.2 (Fig. 3). Compared with the wild type (*yy*), a total of 98 individual protein spots in the *Yy* and *yy* mutants exhibited the twofold up- or down-regulated variation (Fig. 4). Of which the expression abundance of the 81 proteins varied between the *YY* and the *yy* lines, and 41 expressed differently between the *Yy* and the *yy* lines.

**Fig. 5:**
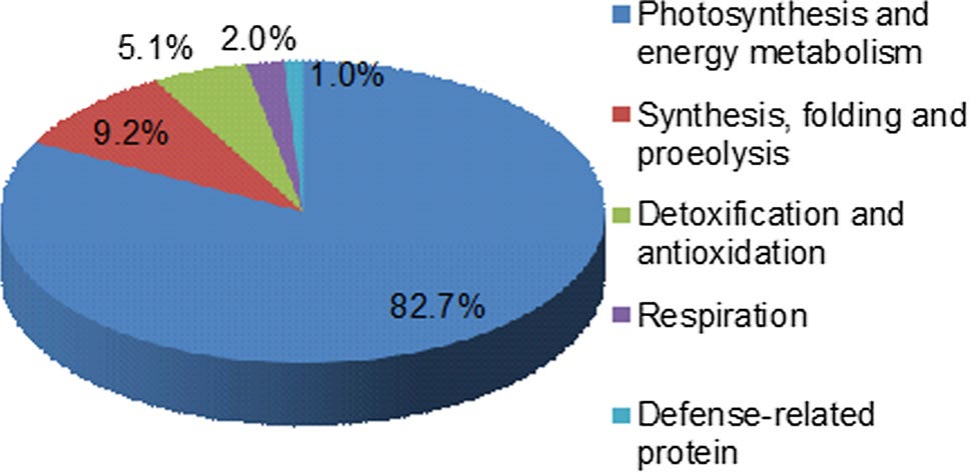
Functional group classification of the identified DEPs in sesame leaf color mutant *Siyl*-*1*. The functional classification of the DEPs is performed based on the KEGG pathway analysis dataset (http://www.kegg.jp/kegg/pathway.html)

Furthermore, we digested the 98 protein spots with trypsin and performed the MALDI-TOF-TOF analysis. The 98 proteins were ultimately screened using the NCBInr (nonredundant) database (Fig. 3; Table 4S). The Kyoto Encyclopedia of Genes and Genomes (KEGG, http://www.kegg.jp/kegg/pathway.html) analysis results reflected that the 98 proteins were grouped into 5 functional categories, i.e., photosynthesis and energy metabolism (82.7%), synthesis, folding and proteolysis (9.2%), detoxification and antioxidation (5.1%), respiration (2.0%), and defense-related protein (1.0%) (Fig. 5). The major group was involved in the photosynthesis bioprocess. Among the DEPs, there were 65 down-regulated light-reaction proteins (Table 4S), including oxygen-evolving enhancer protein (OEE), cytochrome b6-f complex (Cyt b6-f), iron-sulfur subunit, ferredoxin-NADP reductase (FNR), chlorophyll a–b binding protein (LHC), and photosystem I reaction center (PSI), etc.

**Fig. 6:**
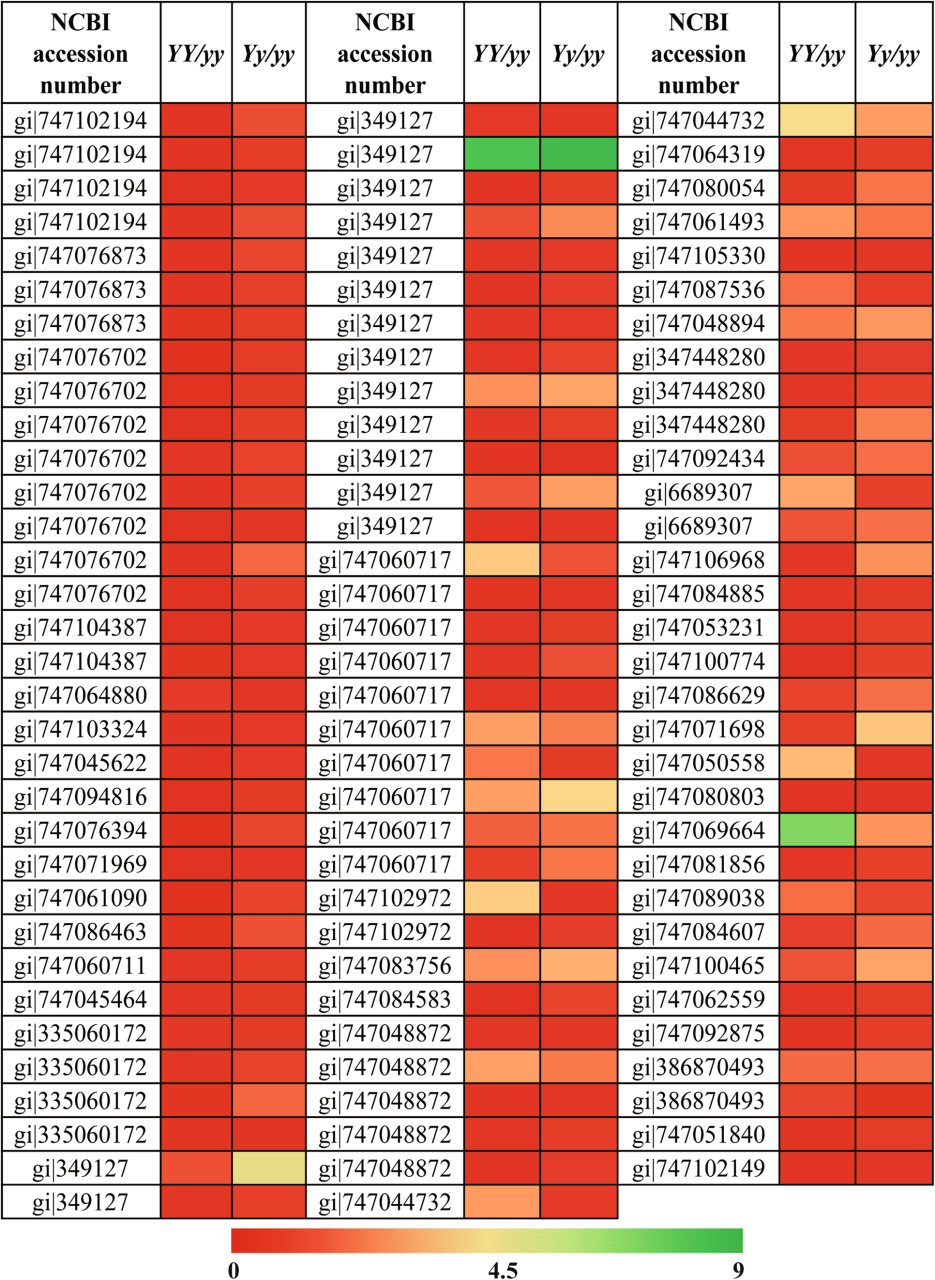
Heat map representation of the 98 DEPs identified in the three genotypes of the mutant *Siyl*-*1* progeny. Color scores are normalized by the log_2_ transformed protein abundance ratio of each spot. Red represents the increased protein abundance, and green represents the decreased protein abundance. *YY*: Homozygous mutant (lethal) with light-yellow leaf color. *Yy*: Heterozygous mutant with yellow-green leaf color. *yy*: Wild type with the normal green leaf color

In addition, we compared the expression patterns of 98 DEPs in the three genotypes according to the heat map (Fig. 6). In the comparison of the *YY* and the *yy* lines, the number of down- and up-regulated proteins was 62 and 19, respectively. For the *Yy* and the *yy* lines, 16 down- and 25 up-regulated proteins were identified. More importantly, there were 11 common DEPs with the down-regulation coordination in the above comparison. Meanwhile, the other 11 common DEPs presented the up-regulated coordination in the two groups (Table 4S).

Further analysis reflected that about half of the DEPs had different molecular masses or isoelectric points (Table 4S). For instance, PSBP1 possessed the four different molecular masses or isoelectric points. We further analyzed the protein–protein interactions and molecular functions of DEPs using STRING 9.0 (Figs. 1S, 2S). The results revealed that the DEPs formed a complicated interaction network, and most of the core interacting proteins were related to the photosynthesis and energy metabolism function (Table 5S). In addition, there were 21 proteins (i.e., ATPQ, PB, LHCA1, LHCA3, LHCA5, GS2, RPS1, FBA2, PSBO2, PSI-P, PETC, GAPA, PSAD-2, RBCL, PSBP-1, CYP38, cpHsc70-1, RPE, TRX-M4, CPN60A, and At5g06290) that individually interacted with more than 15 proteins. For example, the TRX-M4 (thioredoxin-M4) protein had the interaction relationship with 27 proteins. Similarly, GAPA exhibited the interaction with 27 proteins.

### Expression profiles of genes involved in DEPs of *Siyl*-*1*

**Fig. 7:**
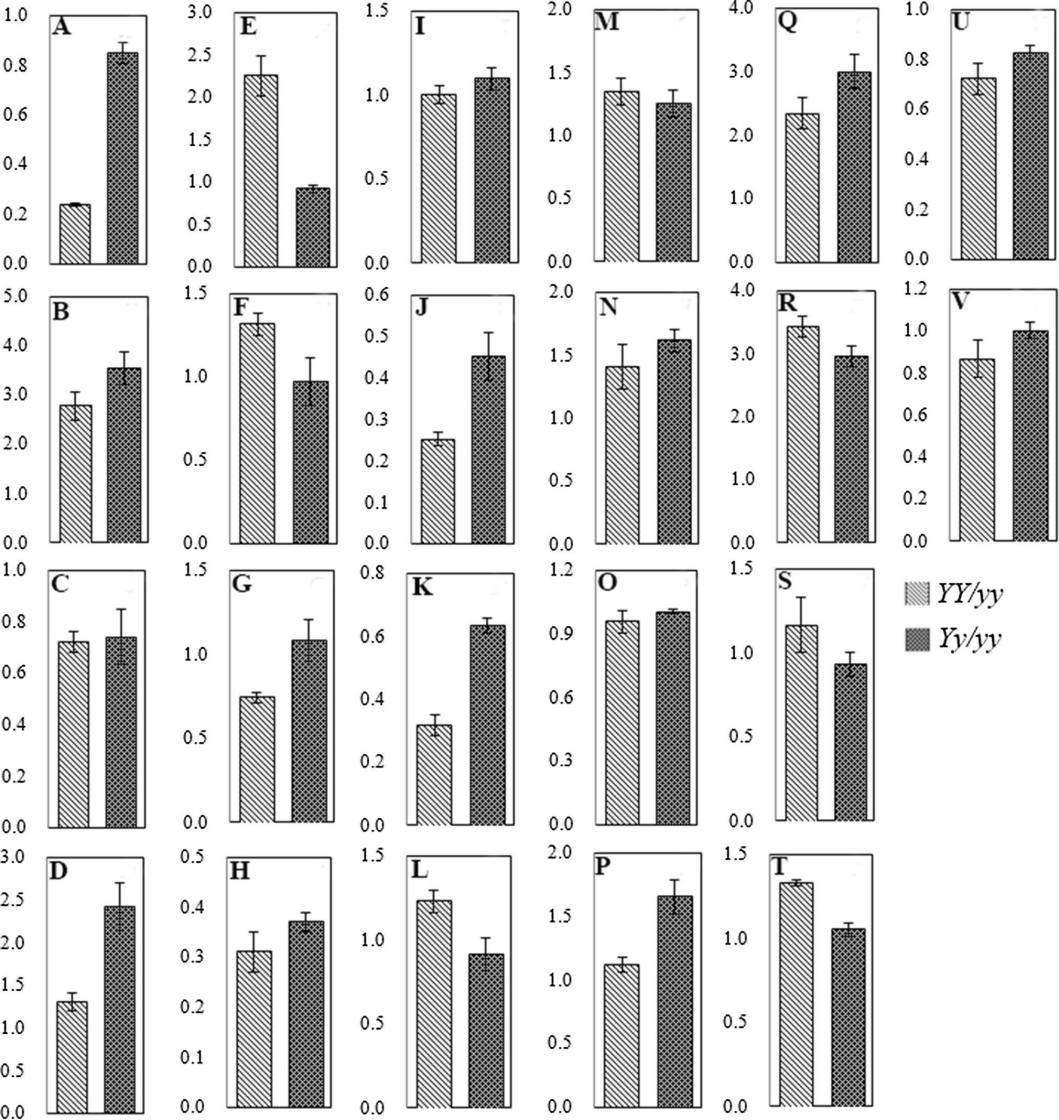
Expression profile analysis of the 22 genes encoding photosynthesis and energy metabolism proteins identified in *Siyl*-*1*. The bar in the column indicates the standard deviation of the transcription level. **a** Oxygen-evolving enhancer protein (OEE); **b** transketolase (TKT); **c** cytochrome b6-f complex iron-sulfur subunit (Cyt b6-f); **d** ferredoxin-NADP reductase (FNR); **e** chlorophyll a-b binding protein CP26 (LHC CP26); **f** chlorophyll a-b binding protein 13 (LHC 13); **g** chlorophyll a-b binding protein 21 (LHC 21); **h** chlorophyll a-b binding protein 8 (LHC 8); **i** chlorophyll a-b binding protein 6 (LHC 6); **j** photosystem I reaction center subunit II (PS I); **k** thylakoid lumenal 15 kDa protein; **l** protein curvature thylakoid; **m** ribulose bisphosphate carboxylase/oxygenase activase; **n** ribulosebis phosphate carboxylase small chain; **o** rubisco large subunit-binding protein subunit alpha; **p** rubisco large subunit-binding protein subunit beta; **q** glyceraldehyde-3-phosphate dehydrogenase (GAPA); **r** Ribose-5-phosphate isomerase (Rib-5-P); **s** triose phosphate isomerase (TPI); **t** fructose-bisphosphate aldolase (FBA); **u** malate dehydrogenase (ME); **v** biotin carboxylase. *YY*: Homozygous mutant (lethal) with light-yellow leaf color. *Yy*: Heterozygous mutant with yellow-green leaf color. *yy*: Wild type with the normal green leaf color

To study the relationship between protein and gene expression in the mutant *Siyl*-*1*, we matched the mRNA sequence with the DEPs using the genome data of sesame in the NCBI database (https://www.ncbi.nlm.nih.gov/). A total of 22 genes were determined involved in photosynthesis and energy metabolism (Fig. 7). qRT-PCR showed that there were 2 different gene expression patterns, compared with the protein expression (Table 4S). A total of 7 (31.8%) genes had the same expression trend with their own proteins, such as OEE (Fig. 7a), transketolase (TKT) (B), Cyt b6-f (C), LHC 21 (G), LHC 8 (H), photosystem I reaction center subunit II (J), and thylakoid luminal 15 kDa protein (K). Meanwhile, the rest 15 genes (68.2%) presented the different trend from their own protein expression level.

## Discussion

Leaf color mutation in plants is common. The mutated genes can be involved in the biosynthesis and degradation of chlorophyll in a direct or indirect manner or the photosynthesis of plants (Wu et al. 2018a, b). In this study, we analyzed the genetic, cytological, and proteomic characters of the leaf color mutant *Siyl*-*1* in sesame for the first time.

### Genetic analysis

Till now many leaf color mutants were found in rice (Huang et al. 2017a), barley (Qin et al. 2015), soybean (Zou et al. 2003), maize (Sawers et al. 2006), and wheat (Zhang et al. 2017). Most of the leaf color mutants are controlled by a single recessive nuclear gene (Zou et al. 2003; Ansari et al. 2013; Deng et al. 2014). For a few leaf color mutants in which the traits are controlled by a cytoplasmic gene, the mutants often exhibit the cytoplasmic inheritance and display a mosaic phenomenon in the developmental process (Chia et al. 1986). In our study, we identified that the yellow leaf trait in the mutant *Siyl*-*1* was controlled by a nuclear gene and displayed incompletely dominance heredity. A yellow-green leaf mutant in wheat was found controlled by a novel single incompletely dominant gene (*Y1718*) on chromosome 2BS (Zhang et al. 2017). In maize, the olivine leaf phenotype also was controlled by a semi-dominant gene (*ZmCHLI*) encoding I subunit of magnesium chelatase (Mg-chelatase) (Sawers et al. 2006). In barley, the three semi-dominance mutations with leaf yellowing phenotype are caused by the deficiency of Xantha-h^clo125^, Xantha-h^clo157^, and Xantha-h^clo161^ gene, respectively (Hansson et al. 2002). In tobacco, a chlorina mutant was also controlled by a semi-dominant *Sulfur* gene encoding the subunit of Mg-chelatase (Fitzmaurice et al. 1999). And offspring of the above mentioned incompletely dominant yellow-green mutant had three different phenotypes: light-yellow (*YY*), yellow-green (*Yy*), and normal green (*yy*) leaf color, similar to our identified offspring phenotypes of yellow–green mutant *Siyl*-*1* in sesame. Moreover, we further analyzed the mutation of above genes resulting in deficient of proteins in the yellow-green mutants, which further resulted in the inhibition of chlorophyll synthesis. However, the proteins have not been identified in our proteomics analysis results. The reason is most likely due to a new incompletely dominant yellow-green gene *Siyl*-*1* identified in yellow-green leaf mutant of sesame and need to be studied further.

### Analysis of pigment content and chloroplast morphology

The chloroplast is an essential organelle for photosynthesis in plants that contains chlorophyll and carotenoids. In the present study, the content of chlorophyll in the mutant was significantly lower than that in the wild type. This is a common phenomenon in leaf color mutants (Yang et al. 2011). In general, the morphological characteristics of the normal chloroplasts show an elliptic or approximately elliptic shape, and the size and the number are stable. In the *Siyl*-*1* mutant, the shape and internal structure of the chloroplast changed dramatically (Fig. 2). These abnormal changes indicated that chloroplast development was restricted. The chlorophyll biosynthesis was abnormal, and leaf color was affected. The similar results also showed in other crops (Wu et al. 2014, 2018a, b; Miura et al. 2007). For example, the chloroplast is underdeveloped in a wheat yellow leaf color mutant (Wu et al. 2018a, b; Zhang et al. 2017). The morphology and structure of the chloroplasts are changed in Arabidopsis yellow variegated mutants caused by the loss of *FtsH2* gene encoding ATP-dependent metalloprotease (Miura et al. 2007). In a rice chlorophyll-deficient mutant, the development of chloroplasts significantly delayed (Wu et al. 2014). Similar to the above leaf color mutants, the chloroplast development and the chlorophyll content in the mutant *Siyl*-*1* was significantly affected. These results indicate that the yellow leaf color mutants in leaves are closely related to chloroplast development and chlorophyll synthesis.

### Functional analysis of DEPs

Comparative proteomics analysis provides the qualitative and quantitative expression profile of the differential proteins and aids in developing a systematic comprehension of the underlying the process of regulation. In this study, a total of 98 DEPs were identified using the proteomics approach. The different protein expression profiles in the three genotypes of the *Siyl*-*1* progeny revealed that the leaf color mutagenesis influenced the accumulation and expression of these proteins. In addition, approximately half of the DEPs were detected in multiple spots with different molecular masses or isoelectric points, which implied the existence of posttranslational modifications and isoforms (Zadraznik et al. 2013). 82.7% of the DEPs were involved in photosynthesis and energy metabolism. 76.5% of the DEPs were down-regulated in the *YY/yy* comparison, which lead to the variation of leaf chlorophyll content and cell structure in the mutant. In the present study, the down-regulated photosynthesis-related proteins (OEE, Cyt b6-f, FNR, LHC and PS I) mainly were involved in photosystem I and II and located on the photo membrane and played an important role in photosynthetic electron transport (Table 3S).

OEEs are components of the photosystem II (PSII) reaction center and promote the activation of oxygen, which can effectively organize the transfer of protons and electrons among the photosynthetic electron transport chain, receptors, and donors (Brudvig and Crabtree 1986). OEE1 is a manganese-stabilizing protein required for PSII core assembly/stability, and OEE2 is responsible for catalyzing the splitting of water (Yang et al. 2003; Yi et al. 2005). Based on our proteomic analysis, both OEE1 (1–7) and OEE2 (8–15) significantly decreased in *YY*, compared to wild type. These results indicated that the ability of transferring protons and electrons was strongly suppressed in *Siyl*-*1*.

The LHC is an important component of the light harvesting complex in the thylakoid membrane, because the protein can capture and transduce light energy, increasing the light energy capture efficiency and adjusting the light energy distribution (Xu et al. 2012). All the chlorophyll-binding proteins decreased in the *NYC1*-like mutant (Sato et al. 2018). The chlorina mutant of barley lacks chlorophyll b and the thylakoid membrane polypeptide (Bellemare et al. 1982). When stay-green is transiently overexpressed in the Arabidopsis mutant, both chlorophyll a and b are degraded, and an *NYC1*-deficient mutant did not degrade chlorophyll b and LHCII under the overexpression of *SGR* (Shimoda et al. 2016). In this study, the expression of the several chlorophyll a-b binding proteins (20–24) significantly decreased in *YY*, compared to wild type plants. Thus, we speculated that these different proteins be related to the ability of capturing light in *Siyl*-*1*.

The Cyt b6-f acts as a bridge to PSI (plastocyanin: GAPOR) via electron transfer (Haley and Bogorad 1989). The activity of the light-harvesting complex of photosystem II kinase is regulated by a Cyt b6-f component(s), which responds to the balance of electron flow from photosystem II to photosystem I via the plastoquinone pool (Gal et al. 1988). Thylakoids could not be phosphorylated under any experimental conditions in Cyt b6-f (Gal et al. 1987). FNR located in the thylakoid membrane can catalyze electron transfer from iron-sulfur, leading to NADP^+^ (Carrillo and Ceccarelli 2003). When the *FNR* gene is mutated, the chlorophyll level is also reduced in the mutant and a yellow-green leaf phenotype is exhibited (Li et al. 2015). In this study, the Cyt b6-f (16, 17), FNR (19), PSI protein (25) and thylakoid protein (26, 27) were down expressed significantly in *YY*, compared to wild type plants. The change in the thylakoid structure might cause the PSI protein deactivation, which further resulted in the loss of electron transfer function of Cyt b6-f.

TKT is a key enzyme in the Calvin cycle of photosynthesis and the pentose phosphate pathway in all organisms. The low amounts of TKT should repress the efficiency of the Calvin cycle and the pentose phosphate pathway, hindering the photosynthesis rate and resulting in plant death (Zhao et al. 2018). Thus, TKT plays an important role in plant defense and growth. However, there are different opinions about the function of *TKT* in the plant. Previous studies showed that *TKL*-overexpressed tobacco plants exhibited chlorosis and plant growth inhibition (Khozaei et al. 2015). Moreover, plant growth and the Chl contents did not changed in the *TKL*-overexpressed rice plants (Suzuki et al. 2017). At present, the reason for this difference is still unclear. Interestingly, the protein expression level of TKT increased in *YY*, which presented the similar protein expression profiles in Khozaei’s report. Thus, the above evidence proved that transketolase probably was indirectly related to photosynthesis. The mechanism of TKT protein expression, along with expression mechanisms of other similar proteins such as biotin carboxylase and triosephosphate isomerase (TPI), was worthy of investigation.

Other DEPs involved in the Calvin cycle, energy conversion, and synthesis, among others, had substantial differences in their expression profiles. For example, 1,5-diphosphate ribulose carboxylase/oxygenase (Rubisco), glyceraldehyde-3-phosphase dehydrogenase (GAPA), and adenosine triphosphate synthase (ATPase). The physiological and molecular mechanisms of these processes require further investigation.

### Expression levels of genes related to DEPs

Leaf color mutants are valuable materials for understanding chloroplast development, chlorophyll biosynthesis and metabolism and photosynthesis, and gene functional analysis. The biosynthesis and expression of genes might be reprogrammed during the mutation (Liu et al. 2011). For instance, in a nuclear mutant of *C. reinhardtii*, the expression level of oxygen-evolving activity was reduced due to the absence of OEE2 polypeptides (Mayfield et al. 1987). The expression level of *TKT* was induced in TKT-overexpressed tobacco plants which exhibited phenotype of chlorosis and inhibition of plant growth (Khozaei et al. 2015). FNR does not influence the distribution of excitation energy between photosystem II and photosystem I, and it does not impact the occurrence of ‘light-state transitions’ (van Thor et al. 1999). Moreover, in Arabidopsis, the expression level of *LHC* also increased in the Mybs1 mutant, whereas the Mybs2 mutant exhibited the decreased expression character (Chen et al. 2016). In addition, expression profile of the Rubisco large subunit (RbcL) also increased in the pale plants, compared with the qRT-treated plants. The RbcL protein promotes the accumulation of Rubisco (Huang et al. 2015). For *D. salina*, glyceraldehyde-3-phosphate dehydrogenase (GAPDH) was reduced to 41.2% and 67.4% in the transformant G1 and G2 of the wild type, respectively (Jia et al. 2009). The mutant plant contained DEPs, and differential gene expression levels were evident between the wild type and the mutants. In this report, the expression trend was consistent with previous studies on OEE (Mayfield et al. 1987) and TKT (Khozaei et al. 2015). Expression level of *GAPDH* gene was opposite to the results shown in Jia et al. (2009). However, the expression of the chlorophyll a/b binding protein (Chen et al. 2016) and Rubisco (Huang et al. 2015) exhibited the increasing and decreasing trend, respectively. These results suggested that the greater influence of gene expression in mutation plants might resulted from the different mRNA expression level of each gene. In addition, our results also reflected that the transcription level and protein level of some genes showed an opposite trend in *Yy/yy*, compared to *YY/yy*. For example, *LHC CP26*, *LHC 6*, *protein curvature thylakoid 1B*, *TPI*, and *FBA* genes less abundant for protein expression but higher expression for translation levels. This reason for the less relevance between the mRNA and the protein level might be related to the post-translational modifications and/or regulation, such as protein phosphorylation, protein acetylation, and protein degradation (Pradet-Balade et al. 2001).

## Conclusions

Genetic, cytological, and proteomic analyses of the sesame yellow leaf mutant, *Siyl*-*1* (*Yy*), were performed for the first time. The yellow leaf mutation (*Yy*) is controlled by an incompletely dominant gene. The chlorophyll content dramatically decreased, and the ultrastructure of the leaf chloroplasts was significantly altered in *Siyl*-*1*, compared to the wild type. The proteomic analysis reflected the expression profiles of the 98 DEPs in the *YY* and *Yy* genotypes. Most of the DEPs were associated with the photosynthesis and chlorophyll biosynthesis pathways. In addition, among the identified proteins, the OEE, Cyt b6-f, CHL, PS I center, and thylakoid proteins were found down-regulated in the yellow-green leaf color mutant. The proteins have important functions in response to the morphogenesis of chloroplasts, photosynthetic electron transport, and light absorption. The changes in the biological processes may be associated with the yellow leaf phenotype. Overall, our findings provided the basis for further exploration underlying the regulation mechanisms of leaf color formation in sesame.

## Electronic supplementary material

Below is the link to the electronic supplementary material.

Supplementary material 1 (TIFF 45 kb)

Supplementary material 2 (TIFF 262 kb)

Supplementary material 3 (DOCX 17 kb)

Supplementary material 4 (DOCX 20 kb)

Supplementary material 5 (DOCX 16 kb)

Supplementary material 6 (DOC 203 kb)

Supplementary material 7 (DOC 99 kb)

## Abbreviations

ATPase: Adenosine triphosphate synthase
ATPQ: ATP synthase subunit d
Chl: Chlorophyll
Cm: Chloroplast outer membrane
Ct: Chloroplast
CYP 38: Cyclophilin 38
Cyt b6-f: Cytochrome b6-f complex iron-sulfur subunit
2-DE: Two-dimensional gel electrophoresis
DEPs: Differentially expressed proteins
EMS: Ethylmethylsulfone
FBA: Fructose-bisphosphate aldolase
FNR: Ferredoxin-NADP reductase
GAPA: Glyceraldehyde-3-phosphate dehydrogenase A
GAPDH: Glyceraldehyde-3-phosphate dehydrogenase
Gr: Grana
GS2: Glutamine synthetase
LHCA1: Photosystem I light harvesting complex gene 1
LHC: Chlorophyll a-b binding protein
ME: Malate dehydrogenase
MP: The number of identified peptides for each protein
OEE: Oxygen-evolving enhancer protein
Og: Osmiophilic globule
PETC: Photosynthetic electron transfer C
PSAD-2: Photosystem I subunit D-2
PSBO2: Photosystem II subunit O-2
PSBP-1: Photosystem II subunit P-1
PS I: Photosystem I reaction center
PSI-P: Photosystem I P subunit
RbcL: Rubisco large subunit
Rib-5-P: Ribose-5-phosphate isomerase
RPS1: Ribosomal protein S1
Rubisco: 1,5-diphosphate ribulose carboxylase/oxygenase
SC: The sequence coverage of identified proteins
TCA: Trichloroacetic acid
Th: Thylakoid
TKT: Transketolase
TPI: Triosephosphate isomerase
Ve: Vesicle
*YY*: Light yellow
*yy*: Normal green
*Yy*: Yellow–green
