# Supplementary material for: Cytological, genetic, and proteomic analysis of a sesame (Sesamum indicum L.) mutant Siyl-1 with yellow–green leaf color
Source: Genes Genomics. 2019 Nov 1;42(1):25–39. doi: 10.1007/s13258-019-00876-w (PMC6942039; doi:10.1007/s13258-019-00876-w)
Supplement: Supplementary file 3 — Supplementary material 3 (DOCX 17 kb) [file 13258_2019_876_MOESM3_ESM.docx]

**Table 1S.** Primer sequences used for qRT-PCR of genes involved in photosynthesis and energy metabolism in *Siyl-1*

| Gene | Oligonucleotide sequences of forward primers | Oligonucleotide sequences of reverse primers |
| --- | --- | --- |
| Oxygen-evolving enhancer protein | AGATTGAGGGTCCTTTTGTGGTA | CGAGCTGCTTGATGGTGAAGA |
| Transketolase | TTCACAGCCCTGGCTTCATT | CTTGCTAAATGCTCAATGGGC |
| Cytochrome b6-f complex iron-sulfur subunit | TGGAGAGCGACAGAACACTTG | CTCTTCCTTGATTGTTGTATTGGG |
| Ferredoxin—NADP reductase | ATCATTATCTCGTCGATTCCCT | AGAACAAACCAACGCCCAA |
| Chlorophyll a-b binding protein CP26 | TCAATTGGCGTGTCCGAGA | TTGGACTTAGGAGCAGGTGCA |
| Chlorophyll a-b binding protein 13 | AGCCCTGTTCCTGGCAAAG | CTTGGGACCATTCTCAGCTCA |
| Chlorophyll a-b binding protein 21 | AGTTATTCTGATGGGAGCTGTTGA | AGCCCCAACGGGTCAAA |
| Chlorophyll a-b binding protein 8 | TGGTGCTATGGTTCCTGCTG | TTTCGGACCCTGAAGGCA |
| Chlorophyll a-b binding protein 6 | GGAATTCTTGTACCGGAGGCT | GCCACGAATTCAATCACCAAA |
| Photosystem I reaction center subunit II | CCACCCTCTCAGCAGCAAA | TTCGGGCTCTTTGGTCG |
| Thylakoid lumenal 15kDa protein | AACCCTTGCCAAGCTGAACA | AGAGGTACTCCCGTTGGTCAT |
| Protein curvature thylakoid | ATTGGTCTTGGAAAGGTAGGTGTA | TCTTCCTCCGCCCTCCTTA |
| Ribulose bisphosphate carboxylase/oxygenase activase | TTGACTGTATATTGCGTGGTTCCT | GGAGAGCCTGCGAAATTGAT |
| Ribulose bisphosphate carboxylase small chain | TGGCTTGTAGGCAATGAAACT | ACGAGGCTGTAAGGGCAC |
| RuBisCO large subunit-binding protein subunit alpha | TTGTGTAGTGAGCACCATTCCA | TGAATGGGAGTTCGGTTACAAT |
| RuBisCO large subunit-binding protein subunit beta | GCTGAGCCATCCTTGTTGAA | CAGCAGGGTGATGGATGATAA |
| Glyceraldehyde-3-phosphate dehydrogenase | ACGAAAGGAGCAAGGCAGTT | GAGTGTTTGTGGACAGAGAAGGT |
| Probable ribose-5-phosphate isomerase | TCACTTGCTCTTGATACTCACTCCA | TCAAAACTCCCATAAAGGACTCAG |
| Triosephosphate isomerase | CAATGGCCTTAGTTTGTGCA | GTAATTGCTTGTATTGGCGAGA |
| Fructose-bisphosphate aldolase | ACAATGCCCTGATCAACAAGTA | GGACCCTTCTCGTTACAGCT |
| Malate dehydrogenase | TCCAAGGACCCAGTCACGAA | CATAACTACTGTCCAGCAGCGTG |
| Biotin carboxylase | ACGTTTGCCAGATTTATGACCA | GGAGGACAACAGGAATCAGGA |
