# Supplementary material for: Cytological, genetic, and proteomic analysis of a sesame (Sesamum indicum L.) mutant Siyl-1 with yellow–green leaf color
Source: Genes Genomics. 2019 Nov 1;42(1):25–39. doi: 10.1007/s13258-019-00876-w (PMC6942039; doi:10.1007/s13258-019-00876-w)
Supplement: Supplementary file 4 — Supplementary material 4 (DOCX 20 kb) [file 13258_2019_876_MOESM4_ESM.docx]

**Table 2S.** Genetic analyses of the yellow leaf trait in *Siyl-1*

| **Cross combination**  **(Female** ×**Male)** | **Number of F_1_** |  | | **Phenotype separation** | | | **χ^2^** | ***p*** |
| --- | --- | --- | --- | --- | --- | --- | --- | --- |
|  |  | **Dead with pale yellowleaf**  **(*YY*)** | **Yellow green leaf**  **(*Yy*)** | | **Green leaf**  **(*yy*)** | **Expected ratio** |  |  |
| *Siyl-1* (*Yy*) ×*Siyl-1* (*Yy*) | 4607 | 1151 | 2328 | | 1128 | 1:2:1 | 0.751 | ＞0.05 |
| *Siyl-1* (*Yy*) ×*Yuzhi 4* (*yy*) | 59 | 0 | 28 | | 31 | 1:1 | 0.068 | ＞0.05 |
| *Yuzhi 4* (*yy*) ×*Siyl-1* (*Yy*) | 92 | 0 | 44 | | 48 | 1:1 | 0.098 | ＞0.05 |
| *Siyl-1* (*Yy*)×*Zhengtaizhi 1* (*yy*) | 245 | 0 | 119 | | 126 | 1:1 | 0.147 | ＞0.05 |
| *Zhengtaizhi 1* (*yy*) ×*Siyl-1* (*Yy*) | 192 | 0 | 89 | | 103 | 1:1 | 0.880 | ＞0.05 |
| *Siyl-1* (*Yy*) ×*Zhengheizhi 1* (*yy*) | 185 | 0 | 102 | | 83 | 1:1 | 1.751 | ＞0.05 |
| *Zhengheizhi 1* (*yy*)×*Siyl-1* (*Yy*) | 286 | 0 | 138 | | 148 | 1:1 | 0.283 | ＞0.05 |
