# Supplementary material for: Cytological, genetic, and proteomic analysis of a sesame (Sesamum indicum L.) mutant Siyl-1 with yellow–green leaf color
Source: Genes Genomics. 2019 Nov 1;42(1):25–39. doi: 10.1007/s13258-019-00876-w (PMC6942039; doi:10.1007/s13258-019-00876-w)
Supplement: Supplementary file 5 — Supplementary material 5 (DOCX 16 kb) [file 13258_2019_876_MOESM5_ESM.docx]

**Table 3S.** Proteomics comparison of the mutant types (*YY* and *Yy*) and the wild type (*yy*) of *Siyl-1* using 2D gels. YY, light-yellow (lethal); Yy, yellow-green; and yy, normal green.

| **Item** | ***YY*** | ***Yy*** | ***yy*** |
| --- | --- | --- | --- |
| Protein quality (mg) | 4.5 | 4.5 | 4.5 |
| Number of protein spots | 518±15 | 521±15 | 535±15 |
| Number of different protein spots (%) | 74 (14.3) | 87 (16.7) | 47 (8.8) |
| Numberof up-accumulated spots | 60 | 63 | 31 |
| Number of down-accumulated spots | 14 | 24 | 16 |
