# Supplementary material for: Cytological, genetic, and proteomic analysis of a sesame (Sesamum indicum L.) mutant Siyl-1 with yellow–green leaf color
Source: Genes Genomics. 2019 Nov 1;42(1):25–39. doi: 10.1007/s13258-019-00876-w (PMC6942039; doi:10.1007/s13258-019-00876-w)
Supplement: Supplementary file 6 — Supplementary material 6 (DOC 203 kb) [file 13258_2019_876_MOESM6_ESM.doc]

**Table 4S.** Identification of differentially expressed proteins in the *yy*, *Yy* and *YY*.

| **Serial number** | **Spot no.^a^** | **NCBI accession number^b^** | **Protein name ^c^** | **Protein score^d^** | **SC** | **MP^f^** | **Mutant /wild type^g^** | | **Teoretical** | **Experimental** |
| --- | --- | --- | --- | --- | --- | --- | --- | --- | --- | --- |
|  |  |  |  |  | **(%)^e^** |  | ***YY/yy^i^*** | ***Yy/yy^j^*** | **TMr/Tpl^k^** | **EMr/Epl^l^** |
| **Photosynthesis and energy metabolism** | | | |  |  |  |  |  |  |  |
| 1 | SSP 4501 | gi|747102194 | PREDICTED: oxygen-evolving enhancer protein 1, chloroplastic [Sesamum indicum] | 257 | 17 | 3 | 0.31±0.01^h)^ | 1.33±0.06 | 42.9/7.60 | 34.9/5.46 |
| 2 | SSP 3317 | gi|747102194 | PREDICTED: oxygen-evolving enhancer protein 1, chloroplastic [Sesamum indicum] | 74 | 6 | 1 | 0.26±0.03^h)^ | 0.74±0.08 | 42.9/7.60 | 23.6/5.03 |
| 3 | SSP 0501 | gi|747102194 | PREDICTED: oxygen-evolving enhancer protein 1, chloroplastic [Sesamum indicum] | 82 | 6 | 1 | 0.10±0.02^h)^ | 0.58±0.06 | 42.9/7.60 | 31.6/4.65 |
| 4 | SSP 2601 | gi|747102194 | PREDICTED: oxygen-evolving enhancer protein 1, chloroplastic [Sesamum indicum] | 153 | 11 | 2 | 0.28±0.05^h)^ | 1.30±0.18 | 42.9/7.60 | 39.4/5.05 |
| 5 | SSP 4313 | gi|747076873 | PREDICTED: oxygen-evolving enhancer protein 1, chloroplastic-like [Sesamum indicum] | 60 | 6 | 1 | 0.29±0.05^h)^ | 1.11±0.09 | 34.3/6.53 | 24.0/5.22 |
| 6 | SSP 5311 | gi|747076873 | PREDICTED: oxygen-evolving enhancer protein 1, chloroplastic-like [Sesamum indicum] | 133 | 12 | 3 | 0.27±0.04^h)^ | 0.86±0.06 | 43.0/6.78 | 33.9/5.48 |
| 7 | SSP 2316 | gi|747076873 | PREDICTED: oxygen-evolving enhancer protein 1, chloroplastic-like [Sesamum indicum] | 297 | 19 | 4 | 0.18±0.02^h)^ | 0.72±0.08 | 64.5/5.55 | 30.7/4.79 |
| 8 | SSP 6306 | gi|747076702 | PREDICTED: oxygen-evolving enhancer protein 2-1, chloroplastic-like [Sesamum indicum] | 33 | 5 | 1 | 0.03±0.00^h)^ | 0.79±0.16 | 51.9/5.72 | 32.8/5.88 |
| 9 | SSP 1306 | gi|747076702 | PREDICTED: oxygen-evolving enhancer protein 2-1, chloroplastic-like [Sesamum indicum] | 33 | 5 | 1 | 0.06±0.01^h)^ | 0.67±0.03 | 44.5/5.78 | 16.2/4.70 |
| 10 | SSP 5310 | gi|747076702 | PREDICTED: oxygen-evolving enhancer protein 2-1, chloroplastic-like [Sesamum indicum] | 76 | 5 | 1 | 0.10±0.01^h)^ | 0.85±0.14 | 29.4/5.89 | 27.1/5.47 |

| **Table 4S.** Continued | | |  | |  | |  | | |  |  | |  | |  | |  | |  |
| --- | --- | --- | --- | --- | --- | --- | --- | --- | --- | --- | --- | --- | --- | --- | --- | --- | --- | --- | --- |
| **Serial number** | **Spot no.^a^** | **NCBI accession number^b^** | **Protein name ^c^** | | **Protein score^d^** | | **SC** | | | **MP^f^** | **Mutant /wild type^g^** | | | | **Teoretical** | | **Experimental** | |  |
|  |  |  |  |  |  |  | **(%)^e^** | | |  | ***YY/yy^i^*** | | ***Yy/yy^j^*** | | **TMr/Tpl^i^** | | **EMr/Epl^j^** | |  |
| 11 | SSP 6406 | gi|747076702 | PREDICTED: oxygen-evolving enhancer protein 2-1, chloroplastic-like [Sesamum indicum] | | 102 | | 5 | | | 1 | 0.38±0.06^h)^ | | 1.01±0.20 | | 20.3/8.33 | | 27.9/5.95 | |  |
| 12 | SSP 2405 | gi|747076702 | PREDICTED: oxygen-evolving enhancer protein 2-1, chloroplastic-like [Sesamum indicum] | | 116 | | 5 | | | 1 | 0.21±0.02^h)^ | | 0.98±0.06 | | 20.3/8.33 | | 25.0/5.01 | |  |
| 13 | SSP 6307 | gi|747076702 | PREDICTED: oxygen-evolving enhancer protein 2-1, chloroplastic-like [Sesamum indicum] | | 199 | | 10 | | | 2 | 0.09±0.02^h)^ | | 0.45±0.09^h)^ | | 20.3/8.33 | | 30.3/5.88 | |  |
| 14 | SSP 2309 | gi|747076702 | PREDICTED: oxygen-evolving enhancer protein 2-1, chloroplastic-like [Sesamum indicum] | | 253 | | 16 | | | 3 | 0.21±0.02^h)^ | | 1.96±0.44 | | 27.3/5.72 | | 24.9/4.87 | |  |
| 15 | SSP 2324 | gi|747076702 | PREDICTED: oxygen-evolving enhancer protein 2-1, chloroplastic-like [Sesamum indicum] | | 254 | | 16 | | | 3 | 0.15±0.01^h)^ | | 0.85±0.06 | | 62.8/5.19 | | 25.1/4.70 | |  |
| 16 | SSP 7309 | gi|747104387 | PREDICTED: cytochrome b6-f complex iron-sulfur subunit, chloroplastic, partial [Sesamum indicum] | | 104 | | 13 | | | 2 | 0.08±0.00^h)^ | | 0.59±0.09 | | 35.9/6.54 | | 24.9/6.22 | |  |
| 17 | SSP 8309 | gi|747104387 | PREDICTED: cytochrome b6-f complex iron-sulfur subunit, chloroplastic, partial [Sesamum indicum] | | 106 | | 13 | | | 2 | 0.17±0.01^h)^ | | 0.56±0.03 | | 29.8/8.30 | | 27.9/6.26 | |  |
| 18 | SSP 5406 | gi|747064880 | PREDICTED: cytochrome b6-f complex iron-sulfur subunit 1, chloroplastic-like [Sesamum indicum] | | 153 | | 14 | | | 2 | 0.62±0.08 | | 0.43±0.13^h)^ | | 18.1/8.67 | | 28.9/5.71 | |  |
| 19 | SSP 9401 | gi|747103324 | PREDICTED: ferredoxin--NADP reductase, leaf-type isozyme, chloroplastic [Sesamum indicum] | | 103 | | 5 | | | 1 | 0.22±0.02^h)^ | | 0.52±0.08 | | 59.1/6.88 | | 46.1/6.65 | |  |
| 20 | SSP 5603 | gi|747045622 | PREDICTED: chlorophyll a-b binding protein CP26, chloroplastic [Sesamum indicum] | | 224 | | 14 | | | 3 | 0.11±0.02^h)^ | | 0.59±0.13 | | 50.0/5.07 | | 40.2/5.63 | |  |
|  |  |  |  | |  | |  | | |  |  | |  | |  | |  | |  |
| 21 | SSP 1405 | gi|747094816 | PREDICTED: chlorophyll a-b binding protein 13, chloroplastic [Sesamum indicum] | | 71 | | 13 | | | 2 | 0.10±0.01^h)^ | | 0.68±0.05 | | 61.3/5.14 | | 35.2/4.76 | |  |
| 22 | SSP 3312 | gi|747076394 | PREDICTED: chlorophyll a-b binding protein 21, chloroplastic-like [Sesamum indicum] | | 137 | | 9 | | | 2 | 0.03±0.01^h)^ | | 1.11±0.10 | | 28.3/5.14 | | 43.1/5.07 | |  |
| 23 | SSP 6305 | gi|747071969 | PREDICTED: chlorophyll a-b binding protein 8, chloroplastic-like [Sesamum indicum] | | 108 | | 13 | | | 2 | 0.07±0.01^h)^ | | 0.45±0.02^h)^ | | 29.1/8.53 | | 33.2/5.78 | |  |
| 24 | SSP 6311 | gi|747061090 | PREDICTED: chlorophyll a-b binding protein 6, chloroplastic [Sesamum indicum] | | 150 | | 9 | | | 3 | 0.05±0.01^h)^ | | 1.03±0.08 | | 26.6/6.11 | | 33.2/5.69 | |  |
| 25 | SSP 0310 | gi|747086463 | PREDICTED: photosystem I reaction center subunit II, chloroplastic-like [Sesamum indicum] | | 183 | | 7 | | | 2 | 0.18±0.03^h)^ | | 1.42±0.18 | | 23.6/9.71 | | 24.2/4.60 | |  |
| 26 | SSP 3320 | gi|747060711 | PREDICTED: thylakoid lumenal 15 kDa protein 1, chloroplastic [Sesamum indicum] | | 137 | | 17 | | | 2 | 0.36±0.08^h)^ | | 0.69±0.01 | | 25.3/6.71 | | 21.8/5.28 | |  |
| 27 | SSP 2310 | gi|747045464 | PREDICTED: protein curvature thylakoid 1B, chloroplastic [Sesamum indicum] | | 158 | | 28 | | | 2 | 0.39±0.08^h)^ | | 0.65±0.12 | | 18.1/8.67 | | 20.8/4.81 | |  |
| 28 | SSP 4314 | gi|335060172 | ribulose-1,5-bisphosphate carboxylase/oxygenase large subunit, partial (chloroplast) [Sesamum indicum] | | 88 | | 4 | | | 2 | 0.45±0.01^h)^ | | 0.64±0.11 | | 24.2/7.60 | | 32.6/5.18 | |  |
| 29 | SSP 0503 | gi|335060172 | ribulose-1,5-bisphosphate carboxylase/oxygenase large subunit, partial (chloroplast) [Sesamum indicum] | | 171 | | 6 | | | 3 | 0.40±0.10^h)^ | | 1.01±0.14 | | 41.3/8.38 | | 44.0/4.43 | |  |
| 30 | SSP 2311 | gi|335060172 | ribulose-1,5-bisphosphate carboxylase/oxygenase large subunit, partial (chloroplast) [Sesamum indicum] | | 228 | | 10 | | | 5 | 0.18±0.03^h)^ | | 1.88±0.16 | | 28.6/4.96 | | 28.2/4.96 | |  |
|  |  |  |  | |  | |  | | |  |  | |  | |  | |  | |  |
| 31 | SSP 9309 | gi|335060172 | ribulose-1,5-bisphosphate carboxylase/oxygenase large subunit, partial (chloroplast) [Sesamum indicum] | | 298 | | 11 | | | 4 | 0.27±0.02^h)^ | | 0.26±0.05^h)^ | | 28.3/5.14 | | 43.7/6.56 | |  |
| 32 | SSP 7601 | gi|349127 | ribulose-1,5-bisphosphate carboxylase/oxygenase large subunit, partial (chloroplast) [Sesamum indicum] | | 44 | | 2 | | | 1 | 1.43±0.05 | | 4.84±0.68^h)^ | | 29.1/8.53 | | 42.0/6.41 | |  |
| 33 | SSP 4316 | gi|349127 | ribulose-1,5-bisphosphate carboxylase/oxygenase large subunit, partial (chloroplast) [Sesamum indicum] | | 45 | | 2 | | | 1 | 0.44±0.01^h)^ | | 0.95±0.19 | | 26.6/6.11 | | 24.9/5.42 | |  |
| 34 | SSP 6605 | gi|349127 | ribulose-1,5-bisphosphate carboxylase/oxygenase large subunit, partial (chloroplast) [Sesamum indicum] | | 46 | | 2 | | | 1 | 0.56±0.06 | | 0.35±0.03^h)^ | | 23.6/9.71 | | 44.9/5.80 | |  |
| 35 | SSP 2306 | gi|349127 | ribulose-1,5-bisphosphate carboxylase/oxygenase large subunit, partial (chloroplast) [Sesamum indicum] | | 52 | | 2 | | | 1 | 8.38±0.96^h)^ | | 8.82±0.96^h)^ | | 25.3/6.71 | | 18.5/4.90 | |  |
| 36 | SSP 2503 | gi|349127 | ribulose-1,5-bisphosphate carboxylase/oxygenase large subunit, partial (chloroplast) [Sesamum indicum] | | 70 | | 2 | | | 1 | 0.24±0.05^h)^ | | 0.80±0.05 | | 55.3/5.19 | | 43.1/5.08 | |  |
| 37 | SSP 4408 | gi|349127 | ribulose-1,5-bisphosphate carboxylase/oxygenase large subunit, partial (chloroplast) [Sesamum indicum] | | 85 | | 2 | | | 1 | 1.39±0.26 | | 2.65±0.71^h)^ | | 55.3/5.19 | | 27.1/5.42 | |  |
| 38 | SSP 7303 | gi|349127 | ribulose-1,5-bisphosphate carboxylase/oxygenase large subunit, partial (chloroplast) [Sesamum indicum] | | 85 | | 2 | | | 1 | 0.44±0.04^h)^ | | 0.59±0.08 | | 55.3/5.19 | | 37.2/6.02 | |  |
| 39 | SSP 4310 | gi|349127 | ribulose-1,5-bisphosphate carboxylase/oxygenase large subunit, partial (chloroplast) [Sesamum indicum] | | 87 | | 2 | | | 1 | 0.33±0.05^h)^ | | 0.84±0.02 | | 10.0/5.19 | | 38.9/5.22 | |  |
| 40 | SSP 5319 | gi|349127 | ribulose-1,5-bisphosphate carboxylase/oxygenase large subunit, partial (chloroplast) [Sesamum indicum] | | 107 | | 2 | | | 1 | 0.49±0.10^h)^ | | 0.52±0.02 | | 51.9/5.72 | | 24.6/5.52 | |  |
|  |  |  |  | |  | |  | | |  |  | |  | |  | |  | |  |
| 41 | SSP 0312 | gi|349127 | ribulose-1,5-bisphosphate carboxylase/oxygenase large subunit, partial (chloroplast) [Sesamum indicum] | | 136 | | 3 | | | 2 | 0.35±0.01^h)^ | | 1.02±0.13 | | 51.9/5.72 | | 29.1/4.62 | |  |
| 42 | SSP 9311 | gi|349127 | ribulose-1,5-bisphosphate carboxylase/oxygenase large subunit, partial (chloroplast) [Sesamum indicum] | | 192 | | 9 | | | 4 | 2.75±0.64^h)^ | | 3.18±0.42^h)^ | | 51.9/5.72 | | 42.9/6.64 | |  |
| 43 | SSP 3801 | gi|349127 | ribulose-1,5-bisphosphate carboxylase/oxygenase large subunit, partial (chloroplast) [Sesamum indicum] | | 210 | | 11 | | | 4 | 0.47±0.07^h)^ | | 0.91±0.08 | | 51.9/5.72 | | 60.2/5.21 | |  |
| 44 | SSP 6604 | gi|349127 | ribulose-1,5-bisphosphate carboxylase/oxygenase large subunit, partial (chloroplast) [Sesamum indicum] | | 233 | | 4 | | | 3 | 0.34±0.06^h)^ | | 0.10±0.01^h)^ | | 51.9/5.72 | | 44.1/5.86 | |  |
| 45 | SSP 8314 | gi|349127 | ribulose-1,5-bisphosphate carboxylase/oxygenase large subunit, partial (chloroplast) [Sesamum indicum] | | 279 | | 11 | | | 4 | 1.59±0.32 | | 3.01±0.26^h)^ | | 53.7/5.09 | | 15.2/6.38 | |  |
| 46 | SSP 2802 | gi|349127 | ribulose-1,5-bisphosphate carboxylase/oxygenase large subunit, partial (chloroplast) [Sesamum indicum] | | 317 | | 11 | | | 4 | 0.21±0.05^h)^ | | 0.43±0.06^h)^ | | 53.7/5.09 | | 61.2/4.99 | |  |
| 47 | SSP 5702 | gi|747060717 | PREDICTED: ribulose bisphosphate carboxylase/oxygenase activase, chloroplastic isoform X1 [Sesamum indicum] | | 37 | | 4 | | | 1 | 3.93±0.45^h)^ | | 1.46±0.03 | | 29.3/9.15 | | 58.7/5.62 | |  |
| 48 | SSP 2321 | gi|747060717 | PREDICTED: ribulose bisphosphate carboxylase/oxygenase activase, chloroplastic isoform X1 [Sesamum indicum] | | 45 | | 4 | | | 1 | 0.20±0.04^h)^ | | 0.40±0.15^h)^ | | 19.7/5.48 | | 40.2/4.81 | |  |
| 49 | SSP 5502 | gi|747060717 | PREDICTED: ribulose bisphosphate carboxylase/oxygenase activase, chloroplastic isoform X1 [Sesamum indicum] | | 67 | | 4 | | | 1 | 0.40±0.06^h)^ | | 0.72±0.05 | | 55.4/6.43 | | 43.8/5.60 | |  |
| 50 | SSP 6402 | gi|747060717 | PREDICTED: ribulose bisphosphate carboxylase/oxygenase activase, chloroplastic isoform X1 [Sesamum indicum] | | 125 | | 4 | | | 1 | 0.43±0.09^h)^ | | 1.37±0.19 | | 68.9/5.24 | | 57.2/5.60 | |  |
|  |  |  |  | |  | |  | | |  |  | |  | |  | |  | |  |
| 51 | SSP 2406 | gi|747060717 | PREDICTED: ribulose bisphosphate carboxylase/oxygenase activase, chloroplastic isoform X1 [Sesamum indicum] | | 127 | | 4 | | | 1 | 0.36±0.04^h)^ | | 0.47±0.07^h)^ | | 51.9/5.72 | | 24.1/5.05 | |  |
| 52 | SSP 4315 | gi|747060717 | PREDICTED: ribulose bisphosphate carboxylase/oxygenase activase, chloroplastic isoform X1 [Sesamum indicum] | | 153 | | 6 | | | 3 | 3.03±0.13^h)^ | | 2.40±0.40^h)^ | | 51.9/5.72 | | 33.5/5.26 | |  |
| 53 | SSP 2502 | gi|747060717 | PREDICTED: ribulose bisphosphate carboxylase/oxygenase activase, chloroplastic isoform X1 [Sesamum indicum] | | 177 | | 6 | | | 3 | 2.27±0.43^h)^ | | 0.67±0.10 | | 51.9/5.72 | | 37.2/5.10 | |  |
| 54 | SSP 3106 | gi|747060717 | PREDICTED: ribulose bisphosphate carboxylase/oxygenase activase, chloroplastic isoform X1 [Sesamum indicum] | | 236 | | 14 | | | 3 | 3.05±0.26^h)^ | | 4.28±0.4^h)^ | | 51.9/5.72 | | 16.7/5.28 | |  |
| 55 | SSP 4407 | gi|747060717 | PREDICTED: ribulose bisphosphate carboxylase/oxygenase activase, chloroplastic isoform X1 [Sesamum indicum] | | 299 | | 7 | | | 2 | 1.77±0.27 | | 2.19±0.31^h)^ | | 51.9/5.72 | | 52.8/5.31 | |  |
| 56 | SSP 4505 | gi|747060717 | PREDICTED: ribulose bisphosphate carboxylase/oxygenase activase, chloroplastic isoform X1 [Sesamum indicum] | | 36 | | 4 | | | 1 | 0.87±0.03 | | 2.25±0.51^h)^ | | 75.1/5.23 | | 37.3/5.25 | |  |
| 57 | SSP 7312 | gi|747102972 | PREDICTED: ribulose bisphosphate carboxylase small chain, chloroplastic-like [Sesamum indicum] | | 107 | | 22 | | | 4 | 4.00±0.61^h)^ | | 0.51±0.03 | | 49.2/4.45 | | 27.3/6.35 | |  |
| 58 | SSP 7310 | gi|747102972 | PREDICTED: ribulose bisphosphate carboxylase small chain, chloroplastic-like [Sesamum indicum] | | 125 | | 18 | | | 3 | 0.34±0.06^h)^ | | 0.82±0.09 | | 23.1/7.79 | | 26.7/6.28 | |  |
| 59 | SSP 2906 | gi|747083756 | ruBisCO large subunit-binding protein subunit alpha-like isoform X1 [Sesamum indicum] | | 110 | | 4 | | | 2 | 2.73±0.54^h)^ | | 3.35±0.84^h)^ | | 34.4/7.98 | | 70.5/4.97 | |  |
| 60 | SSP 5103 | gi|747084583 | PREDICTED: ruBisCO large subunit-binding protein subunit beta, chloroplastic [Sesamum indicum] | | 244 | | 7 | | | 3 | 0.10±0.02^h)^ | | 1.03±0.18 | | 31.8/6.76 | | 16.7/6.48 | |  |
|  |  |  |  | |  | |  | | |  |  | |  | |  | |  | |  |
| 61 | SSP 8602 | gi|747048872 | PREDICTED: glyceraldehyde-3-phosphate dehydrogenase A, chloroplastic [Sesamum indicum] | | 87 | | 3 | | | 1 | 0.37±0.10^h)^ | | 0.37±0.11^h)^ | | 65.7/7.20 | | 51.2/6.46 | |  |
| 62 | SSP 9318 | gi|747048872 | PREDICTED: glyceraldehyde-3-phosphate dehydrogenase A, chloroplastic [Sesamum indicum] | | 263 | | 10 | | | 3 | 3.08±0.33^h)^ | | 2.33±0.19^h)^ | | 65.7/7.20 | | 18.2/7.60 | |  |
| 63 | SSP 8406 | gi|747048872 | PREDICTED: glyceraldehyde-3-phosphate dehydrogenase A, chloroplastic [Sesamum indicum] | | 275 | | 16 | | | 4 | 0.30±0.04^h)^ | | 0.41±0.06^h)^ | | 7.90/8.98 | | 48.7/6.95 | |  |
| 64 | SSP 4204 | gi|747048872 | PREDICTED: glyceraldehyde-3-phosphate dehydrogenase A, chloroplastic [Sesamum indicum] | | 73 | | 3 | | | 1 | 0.38±0.05^h)^ | | 0.78±0.26 | | 29.1/7.68 | | 24.3/5.41 | |  |
| 65 | SSP 2702 | gi|747048872 | PREDICTED: glyceraldehyde-3-phosphate dehydrogenase A, chloroplastic [Sesamum indicum] | | 113 | | 7 | | | 2 | 0.27±0.02^h)^ | | 0.75±0.02 | | 29.1/6.74 | | 47.2/4.98 | |  |
| 66 | SSP 7206 | gi|747044732 | PREDICTED: transketolase, chloroplastic [Sesamum indicum] | | 186 | | 6 | | | 4 | 2.97±0.37^h)^ | | 0.62±0.04 | | 19.0/5.74 | | 23.7/6.22 | |  |
| 67 | SSP 1905 | gi|747044732 | PREDICTED: transketolase, chloroplastic [Sesamum indicum] | | 266 | | 7 | | | 4 | 4.44±0.45^h)^ | | 3.00±0.54^h)^ | | 19.0/5.74 | | 67.1/4.91 | |  |
| 68 | SSP 2305 | gi|747064319 | PREDICTED: probable ribose-5-phosphate isomerase 3, chloroplastic [Sesamum indicum] | | 143 | | 8 | | | 2 | 0.40±0.04^h)^ | | 0.77±0.08 | | 52.8/5.53 | | 43.7/4.96 | |  |
| 69 | SSP 2313 | gi|747061493 | PREDICTED: triosephosphate isomerase, cytosolic [Sesamum indicum] | | 38 | | 5 | | | 1 | 2.88±0.63^h)^ | | 2.24±0.28^h)^ | | 50.2/4.99 | | 24.3/4.71 | |  |
| 70 | SSP 6603 | gi|747105330 | PREDICTED: fructose-bisphosphate aldolase 1, chloroplastic-like [Sesamum indicum] | | 95 | | 6 | | | 2 | 0.33±0.06^h)^ | | 0.49±0.06^h)^ | | 41.0/8.58 | | 47.1/5.95 | |  |
| 71 | SSP 7701 | gi|747087536 | PREDICTED: malate dehydrogenase [Sesamum indicum] | | 227 | | 17 | | | 3 | 2.05±0.15^h)^ | | 0.78±0.08 | | 35.9/6.54 | | 46.7/6.24 | |  |
|  |  |  |  | |  | |  | | |  |  | |  | |  | |  | |  |
| 72 | SSP 6404 | gi|347448280 | ATP synthase CF1 alpha subunit (chloroplast) [Sesamum indicum] | | 124 | | 9 | | | 3 | 0.49±0.06^h)^ | | 0.76±0.10 | | 55.3/5.19 | | 58.1/5.74 | |  |
| 73 | SSP 3313 | gi|347448280 | ATP synthase CF1 alpha subunit (chloroplast) [Sesamum indicum] | | 452 | | 12 | | | 5 | 0.49±0.05^h)^ | | 0.94±0.11 | | 55.3/5.19 | | 43.8/5.02 | |  |
| 74 | SSP 4905 | gi|347448280 | ATP synthase CF1 alpha subunit (chloroplast) [Sesamum indicum] | | 516 | | 17 | | | 7 | 0.78±0.16 | | 2.43±0.40^h)^ | | 55.3/5.19 | | 62.1/5.36 | |  |
| 75 | SSP 6204 | gi|747092434 | ATP synthase CF1 alpha subunit (chloroplast) [Sesamum indicum] | | 87 | | 20 | | | 1 | 1.39±0.21 | | 2.06±0.31^h)^ | | 10.0/5.19 | | 18.1/5.58 | |  |
| 76 | SSP 3205 | gi|6689307 | ATP synthase beta subunit, partial [Sesamum indicum] | | 148 | | 7 | | | 2 | 3.19±0.19^h)^ | | 0.96±0.26 | | 53.7/5.09 | | 18.7/5.27 | |  |
| 77 | SSP 7801 | gi|6689307 | ATP synthase beta subunit, partial [Sesamum indicum] | | 132 | | 3 | | | 1 | 1.51±0.15 | | 2.13±0.09^h)^ | | 53.7/5.09 | | 62.4/6.10 | |  |
| 78 | SSP 7906 | gi|747106968 | PREDICTED: ATP synthase subunit alpha, mitochondrial [Sesamum indicum] | | 185 | | 10 | | | 3 | 0.51±0.10 | | 2.77±0.48^h)^ | | 55.4/6.43 | | 64.3/6.43 | |  |
| 79 | SSP 5407 | gi|747084885 | ATP synthase subunit d, mitochondrial [Sesamum indicum] | | 121 | | 15 | | | 2 | 0.48±0.09^h)^ | | 0.64±0.09 | | 19.7/5.48 | | 28.4/5.54 | |  |
| 80 | SSP 8306 | gi|747053231 | PREDICTED: V-type proton ATPase catalytic subunit A [Sesamum indicum] | | 141 | | 4 | | | 2 | 0.42±0.05^h)^ | | 0.97±0.21 | | 68.9/5.24 | | 34.3/6.43 | |  |
| 81 | SSP 5309 | gi|747100465 | PREDICTED: thioredoxin M2, chloroplastic-like [Sesamum indicum] | | 98 | | 22 | | | 1 | 1.49±0.24 | | 3.15±0.50^h)^ | | 7.90/8.98 | | 24.0/5.48 | |  |
| **Synthesis, folding and proeolysis** | | | | |  | | | | | |  | | | |  | |  | |  |
| 82 | SSP 1303 | gi|747100774 | PREDICTED: peptidyl-prolyl cis-trans isomerase CYP38, chloroplastic isoform X1 [Sesamum indicum] | | 390 | 16 | | | 5 | | | 0.11±0.01^h)^ | | 0.97±0.04 | | 50.0/5.07 | | 21.1/4.86 | |
|  |  |  |  | |  |  | | |  | | |  | |  | |  | |  | |
| 83 | SSP 3502 | gi|747086629 | PREDICTED: glutamine synthetase leaf isozyme, chloroplastic [Sesamum indicum] | | 90 | 3 | | | 1 | | | 1.04±0.14 | | 2.11±0.01^h)^ | | 48.0/6.48 | | 35.2/5.26 | |
| 84 | SSP 1204 | gi|747071698 | PREDICTED: 30S ribosomal protein S6 alpha, chloroplastic [Sesamum indicum] | | 94 | 8 | | | 1 | | | 0.95±0.19 | | 3.85±0.79^h)^ | | 23.1/7.79 | | 23.1/4.91 | |
| 85 | SSP 7406 | gi|747050558 | PREDICTED: 30S ribosomal protein S1, chloroplastic-like [Sesamum indicum] | | 359 | 17 | | | 5 | | | 3.60±0.26^h)^ | | 0.42±0.09^h)^ | | 45.5/5.33 | | 31.3/6.11 | |
| 86 | SSP 1106 | gi|747080803 | PREDICTED: stromal 70 kDa heat shock-related protein, chloroplastic-like [Sesamum indicum] | | 413 | 9 | | | 4 | | | 0.27±0.03^h)^ | | 0.30±0.10^h)^ | | 75.1/5.23 | | 15.2/4.81 | |
| 87 | SSP 0207 | gi|747069664 | PREDICTED: 18.8 kDa class II heat shock protein-like [Sesamum indicum] | | 71 | 9 | | | 1 | | | 7.16±0.77^h)^ | | 2.87±0.26^h)^ | | 17.6/5.96 | | 19.6/4.43 | |
| 88 | SSP 0406 | gi|747081856 | PREDICTED: low-temperature-induced cysteine proteinase-like [Sesamum indicum] | | 181 | 9 | | | 3 | | | 0.49±0.09^h)^ | | 0.96±0.09 | | 52.8/5.53 | | 35.1/4.57 | |
| 89 | SSP 0203 | gi|747089038 | PREDICTED: calreticulin [Sesamum indicum] | | 158 | 10 | | | 3 | | | 2.07±0.06^h)^ | | 1.10±0.14 | | 49.2/4.45 | | 24.5/4.42 | |
| 90 | SSP 4801 | gi|747084607 | PREDICTED: tubulin alpha-3 chain [Sesamum indicum] | | 155 | 12 | | | 3 | | | 0.89±0.11 | | 2.01±0.27^h)^ | | 50.2/4.99 | | 60.4/5.41 | |
| **Detoxification and antioxidation** | | | | |  | | | | | |  | | | |  | |  | |  |
| 91 | SSP 1311 | gi|747062559 | PREDICTED: 2-Cys peroxiredoxin BAS1, chloroplastic-like [Sesamum indicum] | | 386 | 23 | | | 4 | | | 0.39±0.10^h)^ | | 0.74±0.08 | | 29.1/7.68 | | 28.6/4.92 | |
| 92 | SSP 2314 | gi|747092875 | PREDICTED: 2-Cys peroxiredoxin BAS1, chloroplastic-like [Sesamum indicum] | | 321 | 23 | | | 4 | | | 0.27±0.03^h)^ | | 0.77±0.06 | | 29.1/6.74 | | 24.1/4.85 | |
| 93 | SSP 7106 | gi|386870493 | polyphenol oxidase [Sesamum indicum] | | 183 | 13 | | | 5 | | | 2.01±0.09^h)^ | | 2.11±0.41^h)^ | | 65.7/7.20 | | 15.1/6.04 | |
| 94 | SSP 8503 | gi|386870493 | polyphenol oxidase [Sesamum indicum] | | 330 | 11 | | | 5 | | | 1.10±0.06 | | 0.18±0.02^h)^ | | 65.7/7.20 | | 39.1/6.45 | |
|  |  |  |  | |  |  | | |  | | |  | |  | |  | |  | |
| 95 | SSP 3318 | gi|747051840 | PREDICTED: haloacid dehalogenase-like hydrolase domain-containing protein At3g48420 [Sesamum indicum] | | 223 | 12 | | | 3 | | | 0.23±0.01^h)^ | | 0.73±0.04 | | 34.4/7.98 | | 43.2/5.00 | |
| **Respiration** | | | | |  | | |  | | |  | |  | |  | |  | |  |
| 96 | SSP 8312 | gi|747048894 | PREDICTED: biotin carboxylase 2, chloroplastic [Sesamum indicum] | 399 | 13 | | | 6 | | | 2.31±0.23^h)^ | | 2.91±0.65^h)^ | | 59.1/6.88 | | 18.1/6.45 | |  |
| 97 | SSP 9313 | gi|747080054 | PREDICTED: ribulose-phosphate 3-epimerase, chloroplastic isoform X1 [Sesamum indicum] | 86 | 5 | | | 1 | | | 0.65±0.29 | | 2.22±0.68^h)^ | | 29.8/8.30 | | 36.0/6.54 | |  |
| **Defense-related protein** | | | | |  | | |  | | |  | |  | |  | |  | |  |
| 98 | SSP 0405 | gi|747102149 | PREDICTED: salicylic acid-binding protein 2-like [Sesamum indicum] | 492 | 24 | | | 5 | | | 0.23±0.02^h)^ | | 0.49±0.06^h)^ | | 31.8/6.76 | | 30.2/4.48 | |  |

a) Numbering corresponds to the 2-DE in Fig.2.

b) Accession number from the NCBInr database.

c) Names and species of the proteins obtained via the MASCOT software from the NCBInr database.

d) MOWSE score probability for the entire protein.

e) The sequence coverage of identified proteins.

f) The number of identified peptide for each protein.

g) The protein abundance ratio (mutant/wild type).

h) Indicates significant (more than 2.0-fold or less than 0.5-fold) difference between mutant and wild type at 0.05 level.

i) *YY* /*yy*

j) *Yy* /*yy*

k) TMr and Tpl are theoretical isoelectric point and theoretical molecular mass, respectively.

l) EMr and Epl are experimental isoelectric point and experimental molecular mass, respectively.
