# Supplementary material for: Cytological, genetic, and proteomic analysis of a sesame (Sesamum indicum L.) mutant Siyl-1 with yellow–green leaf color
Source: Genes Genomics. 2019 Nov 1;42(1):25–39. doi: 10.1007/s13258-019-00876-w (PMC6942039; doi:10.1007/s13258-019-00876-w)
Supplement: Supplementary file 7 — Supplementary material 7 (DOC 99 kb) [file 13258_2019_876_MOESM7_ESM.doc]

**Table 5S.** Interprotein function in a yellow leaf mutant *Siyl-1* in sesame.

| **Protein name** | **Functions** | **The number of interaction proteins** | **Organism** | **Identifier** |
| --- | --- | --- | --- | --- |
| ATPA | ATP synthase subunit alpha; Produces ATP from ADP in the presence of a proton gradient across the membrane. The alpha chain is a regulatory subunit | 13 | Arabidopsis thaliana | ATCG00120.1 |
| ATP1 | ATPase subunit 1; Mitochondrial membrane ATP synthase (F(1)F(0) ATP synthase or Complex V) produces ATP from ADP in the presence of a proton gradient across the membrane which is generated by electron transport complexes of the respiratory chain. F-type ATPases consist of two structural domains, F(1) - containing the extramembraneous catalytic core, and F(0) - containing the membrane proton channel, linked together by a central stalk and a peripheral stalk. | 14 | Arabidopsis thaliana | ATMG01190.1 |
| ATPQ | ATP synthase subunit d; Mitochondrial membrane ATP synthase (F(1)F(0) ATP synthase or Complex V) produces ATP from ADP in the presence of a proton gradient across the membrane which is generated by electron transport complexes of the respiratory chain. F-type ATPases consist of two structural domains, F(1) - containing the extramembraneous catalytic core, and F(0) - containing the membrane proton channel, linked together by a central stalk and a peripheral stalk. | 15 | Arabidopsis thaliana | AT3G52300.1 |
| PB | ATP synthase subunit beta; Produces ATP from ADP in the presence of a proton gradient across the membrane. The catalytic sites are hosted primarily by the beta subunits | 16 | Arabidopsis thaliana | ATCG00480.1 |

| **Table 5S.** Continued | | | | |
| --- | --- | --- | --- | --- |
| **Protein name** | **Functions** | **The number of interaction proteins** | **Organism** | **Identifier** |
| VHA-A | Vacuolar ATP synthase subunit A; Catalytic subunit of the peripheral V1 complex of vacuolar ATPase. V-ATPase vacuolar ATPase is responsible for acidifying a variety of intracellular compartments in eukaryotic cells | 4 | Arabidopsis thaliana | AT1G78900.1 |
| RPT4A | Regulatory particle triple-A ATPase 4A; The 26S protease is involved in the ATP-dependent degradation of ubiquitinated proteins. The regulatory (or ATPase) complex confers ATP dependency and substrate specificity to the 26S complex | 0 | Arabidopsis thaliana | AT5G43010.1 |
| FNR2 | Ferredoxin--NADP+ reductase; Plays a key role in regulating the relative amounts of cyclic and non-cyclic electron flow to meet the demands of the plant for ATP and reducing power | 13 | Arabidopsis thaliana | AT1G20020.1 |
| LHCA1 | Photosystem I light harvesting complex gene 1; The light-harvesting complex (LHC) functions as a light receptor, it captures and delivers excitation energy to photosystems with which it is closely associated | 16 | Arabidopsis thaliana | AT3G54890.1 |
| LHCA3 | Photosystem I light harvesting complex gene 3; The light-harvesting complex (LHC) functions as a light receptor, it captures and delivers excitation energy to photosystems with which it is closely associated, here photosystem I | 15 | Arabidopsis thaliana | AT1G61520.1 |
| LHCB3 | Light-harvesting chlorophyll B-binding protein 3 | 14 | Arabidopsis thaliana | AT5G54270.1 |
| **Table 5S.** Continued | | | | |
| **Protein name** | **Functions** | **The number of interaction proteins** | **Organism** | **Identifier** |
| LHCB5 | Chlorophyll a-b binding protein CP26; The light-harvesting complex (LHC) functions as a light receptor, it captures and delivers excitation energy to photosystems with which it is closely associated | 16 | Arabidopsis thaliana | AT4G10340.1 |
| GS2 | Glutamine synthetase; The light-modulated chloroplast/mitochondrial enzyme, encoded by a nuclear gene and expressed primarily in leaves, is responsible for the reassimilation of the ammonia generated by photorespiration | 22 | Arabidopsis thaliana | AT5G35630.1 |
| RPS1 | Ribosomal protein S1; Required for optimal plastid performance in terms of photosynthesis and growth. Required for the translation of plastid mRNAs (PubMed:22900828). Involved in cellular heat stress response and required for heat tolerance. Required for transcriptional activation of HSFA2 and its target genes in response to heat stress. Plays a critical role in biosynthesis of thylakoid membrane proteins encoded by chloroplast genes (PubMed:22570631) | 18 | Arabidopsis thaliana | AT5G30510.1 |
| FBA2 | Fructose-bisphosphate aldolase 2 | 18 | Arabidopsis thaliana | AT4G38970.1 |
| PSBO2 | Photosystem II subunit O-2; Stabilizes the manganese cluster which is the primary site of water splitting. Regulates dephosphorylation and turnover of the PSII reaction center D1 protein | 19 | Arabidopsis thaliana | AT3G50820.1 |
| **Table 5S.** Continued | | | | |
| **Protein name** | **Functions** | **The number of interaction proteins** | **Organism** | **Identifier** |
| RBCS1A | Ribulose bisphosphate carboxylase small chain 1A; RuBisCO catalyzes two reactions: the carboxylation of D- ribulose 1,5-bisphosphate, the primary event in carbon dioxide fixation, as well as the oxidative fragmentation of the pentose substrate. Both reactions occur simultaneously and in competition at the same active site (By similarity) | 13 | Arabidopsis thaliana | AT1G67090.1 |
| PSI-P | Photosystem I P subunit; Determines thylakoid architecture by inducing membrane curvature | 19 | Arabidopsis thaliana | AT2G46820.1 |
| PETC | Photosynthetic electron transfer C; Essential protein for photoautotrophism. Confers resistance to photo-oxidative damages by contributing to the thermal dissipation of light energy and to lumenal acidification (increase of pH gradient). Component of the cytochrome b6-f complex, which mediates electron transfer between photosystem II (PSII) and photosystem I (PSI), cyclic electron flow around PSI, and state transitions (By similarity) | 25 | Arabidopsis thaliana | AT4G03280.1 |
| GAPA | Glyceraldehyde-3-phosphate dehydrogenase A; Involved in the photosynthetic reductive pentose phosphate pathway (Calvin-Benson cycle). Catalyzes the reduction of 1,3-diphosphoglycerate by NADPH (By similarity) | 27 | Arabidopsis thaliana | AT3G26650.1 |
| **Table 5S.** Continued | | | | |
| **Protein name** | **Functions** | **The number of interaction proteins** | **Organism** | **Identifier** |
| PSAD-2 | Photosystem I subunit D-2; PSAD can form complexes with ferredoxin and ferredoxin- oxidoreductase in photosystem I (PS I) reaction center. PSAD may encode the ferredoxin-docking protein (By similarity) | 15 | Arabidopsis thaliana | AT1G03130.1 |
| RBCL | Ribulose-bisphosphate carboxylases. [Source: TAIR; Acc: ATCG00490]; RuBisCO catalyzes two reactions: the carboxylation of D- ribulose 1,5-bisphosphate, the primary event in carbon dioxide fixation, as well as the oxidative fragmentation of the pentose substrate in the photorespiration process. Both reactions occur simultaneously and in competition at the same active site | 16 | Arabidopsis thaliana | ATCG00490.1 |
| CAB1 | Chlorophyll A/B binding protein 1; The light-harvesting complex (LHC) functions as a light receptor, it captures and delivers excitation energy to photosystems with which it is closely associated | 9 |  | AT1G29930.1 |
| TPI | Triosephosphate isomerase | 12 | Arabidopsis thaliana | AT3G55440.1 |
| EMB3119 | EMBRYO DEFECTIVE 3119; Catalyzes the reversible conversion of ribose-5- phosphate to ribulose 5-phosphate | 11 | Arabidopsis thaliana | AT3G04790.1 |
| PSBP-1 | Photosystem II subunit P-1; May be involved in the regulation of photosystem II | 18 | Arabidopsis thaliana | AT1G06680.1 |
| CYP38 | Cyclophilin 38; Required for the assembly and stabilization of PSII, but has no PPIases activity | 15 | Arabidopsis thaliana | AT3G01480.1 |
| **Table 5S.** Continued | | | | |
| **Protein name** | **Functions** | **The number of interaction proteins** | **Organism** | **Identifier** |
| cpHsc70-1 | Chloroplast heat shock protein 70-1; Acts redundantly with HSP70-7 in the thermotolerance of germinating seeds. Plays an important role in the protein precursor import into chloroplasts | 16 | Arabidopsis thaliana | AT4G24280.1 |
| RPE | D-ribulose-5-phosphate-3-epimerase | 17 | Arabidopsis thaliana | AT5G61410.1 |
| TRX-M4 | Thioredoxin M4; Thiol-disulfide oxidoreductase involved in the redox regulation of enzyme of the oxidative pentose phosphate pathway. Under reducing conditions, inhibits the glucose-6-phosphate dehydrogenase | 27 | Arabidopsis thaliana | AT3G15360.1 |
| PER1 | 1-Cys peroxiredoxin PER1; Antioxidant protein that seems to contribute to the inhibition of germination during stress | 4 | Arabidopsis thaliana | AT1G48130.1 |
| c-NAD-MDH2 | Malate dehydrogenase | 10 | Arabidopsis thaliana | AT5G43330.1 |
| Cpn60beta2 | Chaperonin-60beta2; Involved in protein assisted folding | 13 | Arabidopsis thaliana | AT3G13470.1 |
| CPN60A | Chaperonin-60alpha; Binds RuBisCO small and large subunits and is implicated in the assembly of the enzyme oligomer. Involved in protein assisted folding. Required for proper chloroplast development | 15 | Arabidopsis thaliana | AT2G28000.1 |
| **Table 5S.** Continued | | | | |
| **Protein name** | **Functions** | **The number of interaction proteins** | **Organism** | **Identifier** |
| HSP17.6II | Heat shock protein 17.6-II | 3 | Arabidopsis thaliana | AT5G12020.1 |
| CAC2 | Biotin carboxylase; This protein is a component of the acetyl coenzyme A carboxylase complex; first, biotin carboxylase catalyzes the carboxylation of the carrier protein and then the transcarboxylase transfers the carboxyl group to form malonyl-CoA | 3 | Arabidopsis thaliana | AT5G35360.3 |
| AT3G48420 | Haloacid dehalogenase-like hydrolase (HAD) superfamily protein | 14 | Arabidopsis thaliana | AT3G48420.1 |
| At5g06290 | 2-cysteine peroxiredoxin B; May be an antioxidant enzyme particularly in the developing shoot and photosynthesizing leaf. Involved in the detoxification of alkyl hydroperoxides with reducing equivalents provided through the thioredoxin system (By similarity) | 20 | Arabidopsis thaliana | AT5G06290.1 |
| AT3G60210 | GroES-like family protein | 8 | Arabidopsis thaliana | AT3G60210.1 |
| AT2G45290 | Transketolase; Catalyzes the reversible transfer of a two-carbon ketol group from fructose-6-phosphate or sedoheptulose-7-phosphate to glyceraldehyde-3-phosphate to yield xylulose-5-phosphate and erythrose-4-phosphate or ribose-5-phosphate, respectively (By similarity). Could act as a stress sensor involved in adaptation process | 7 | Arabidopsis thaliana | AT2G45290.1 |
| AT1G64510 | 30S ribosomal protein S6 alpha; Binds together with S18 to 16S ribosomal RNA | 6 | Arabidopsis thaliana | AT1G64510.1 |
| **Table 5S.** Continued | | | | |
| **Protein name** | **Functions** | **The number of interaction proteins** | **Organism** | **Identifier** |
| AT1G12960 | Large subunit ribosomal protein L27Ae | 5 | Arabidopsis thaliana | AT1G12960.1 |
| AT2G44920 | Thylakoid lumenal protein 1 | 2 | Arabidopsis thaliana | AT2G44920.2 |
| CRT1b | Calreticulin-2; Molecular calcium-binding chaperone promoting folding, oligomeric assembly and quality control in the ER via the calreticulin/calnexin cycle. This lectin may interact transiently with almost all of the monoglucosylated glycoproteins that are synthesized in the ER (By similarity) | 0 | Arabidopsis thaliana | AT1G09210.1 |
| TUA3 | Tubulin 3; Tubulin is the major constituent of microtubules. It binds two moles of GTP, one at an exchangeable site on the beta chain and one at a non-exchangeable site on the alpha chain | 1 | Arabidopsis thaliana | AT5G19770.1 |
| MES1 | Methyl esterase 1; Methylesterase shown to have carboxylesterase activity, methyl indole-3-acetic acid (MeIAA) esterase activity, methyl salicylate (MeSA) esterase activity and methyl jasmonate (MeJA) esterase activity in vitro. Required to convert methyl salicylate (MeSA) to salicylic acid (SA) as part of the signal transduction pathways that activate systemic acquired resistance in systemic tissue. MeSA is believed to be an inactive form that needs to be demethylated to exert a biological effect | 0 | Arabidopsis thaliana | AT2G23620.1 |
| **Table 5S.** Continued | | | | |
| **Protein name** | **Functions** | **The number of interaction proteins** | **Organism** | **Identifier** |
| RD21A | Cysteine proteinase RD21a | 0 | Arabidopsis thaliana | AT1G47128.1 |
